# Quantitative sequencing clarifies the role of disruptor taxa, oral microbiota, and strict anaerobes in the human small-intestine microbiome

**DOI:** 10.1186/s40168-021-01162-2

**Published:** 2021-11-02

**Authors:** Jacob T. Barlow, Gabriela Leite, Anna E. Romano, Rashin Sedighi, Christine Chang, Shreya Celly, Ali Rezaie, Ruchi Mathur, Mark Pimentel, Rustem F. Ismagilov

**Affiliations:** 1 Division of Biology and Biological Engineering, California Institute of Technology, 1200 E. California Blvd, Pasadena, CA 91125 USA; 2 Medically Associated Science and Technology (MAST) Program, Cedars-Sinai Medical Center, Los Angeles, CA 90048 USA; 3 Division of Chemistry and Chemical Engineering, California Institute of Technology, 1200 E. California Blvd, Pasadena, CA 91125 USA; 4 Division of Digestive and Liver Diseases, Cedars-Sinai Medical Center, Los Angeles, CA 90048 USA; 5 Division of Endocrinology, Diabetes, and Metabolism, Cedars-Sinai Medical Center, Los Angeles, CA 90048 USA

**Keywords:** Duodenum, Saliva, HACEK, Human small intestinal microbiome, IBS, SIBO, *Enterobacteriaceae*, *Lactobacillus*, Constipation, Bloating

## Abstract

**Background:**

Upper gastrointestinal (GI) disorders and abdominal pain afflict between 12 and 30% of the worldwide population and research suggests these conditions are linked to the gut microbiome. Although large-intestine microbiota have been linked to several GI diseases, the microbiota of the human small intestine and its relation to human disease has been understudied. The small intestine is the major site for immune surveillance in the gut, and compared with the large intestine, it has greater than 100 times the surface area and a thinner and more permeable mucus layer.

**Results:**

Using quantitative sequencing, we evaluated total and taxon-specific absolute microbial loads from 250 duodenal-aspirate samples and 21 paired duodenum-saliva samples from participants in the REIMAGINE study. Log-transformed total microbial loads spanned 5 logs and were normally distributed. Paired saliva-duodenum samples suggested potential transmission of oral microbes to the duodenum, including organisms from the HACEK group. Several taxa, including *Klebsiella, Escherichia*, *Enterococcus*, and *Clostridium*, seemed to displace strict anaerobes common in the duodenum, so we refer to these taxa as disruptors. Disruptor taxa were enriched in samples with high total microbial loads and in individuals with small intestinal bacterial overgrowth (SIBO). Absolute loads of disruptors were associated with more severe GI symptoms, highlighting the value of absolute taxon quantification when studying small-intestine health and function.

**Conclusion:**

This study provides the largest dataset of the absolute abundance of microbiota from the human duodenum to date. The results reveal a clear relationship between the oral microbiota and the duodenal microbiota and suggest an association between the absolute abundance of disruptor taxa, SIBO, and the prevalence of severe GI symptoms.

Video Abstract

**Supplementary Information:**

The online version contains supplementary material available at 10.1186/s40168-021-01162-2.

## Background

Hundreds of studies have linked the human microbiome to specific diseases. In metabolic diseases or gastrointestinal (GI) disorders (e.g., irritable bowel syndrome [IBS], Crohn’s disease, malabsorption) that can cause GI symptoms, such as pain, bloating, and diarrhea, the small intestine instead of the colon may be the primary site of microbial interactions related to disease. Studies have focused on stool primarily for its ease of access and the fact that it has the highest density of microbes out of any human sample type [1]. The stool microbiome has been shown to be a good proxy for the large-intestine microbiome, but is known to differ substantially from the small-intestine microbiome [2, 3]. Compared with the large intestine, the small intestine has several physiological differences that indicate its potential relevance for microbial interactions. The surface area of the small intestine is greater than 100 times that of the large intestine, underlining its role in nutrient absorption. Additionally, the mucus layer of the small intestine is much thinner and more diffuse [4], potentially allowing closer interactions between microbes and the host. Finally, the small intestine is the main site for intestinal immune surveillance by lamina propria dendritic cells [5] and Peyer’s patches [6], contributing to the body’s response to both commensal and pathogenic microbes.

Although mouse studies have been an insightful proxy for understanding the large-intestine microbiome of humans, the coprophagic behavior of mice [7] and many other animal models results in a substantially different small-intestine microbiome compared with humans [8]. For example, the total microbial load of the human small intestine is generally thought to be low, around 10^2^–10^6^ CFU/mL [1], whereas microbial loads in laboratory mice are nearly 10^9^ CFU/mL [8, 9]. In humans, culturable levels above 10^3^–10^5^ CFU/mL from duodenal aspirates are used as the clinical determination of small intestinal bacterial overgrowth (SIBO) [10]. SIBO has been shown to correlate with IBS and GI symptoms such as bloating, constipation, and diarrhea [11, 12]. Physiologically, SIBO has also been linked to slow intestinal transit [13], higher body mass index (BMI) [14], and reduced stomach-acid levels [15]. Standard-of-care treatments for SIBO often include antibiotics and diets designed to reduce the amount of rapidly fermentable products in the small intestine [16]. However, reoccurrence of symptoms after antibiotics is common and adherence to strict diets is often difficult for patients [17]. Only recently has a connection between the relative abundance of specific microbial taxa, generally from the *Enterobacteriaceae* family, and SIBO begun to be uncovered [18].

The difficult nature of sampling most of the gastrointestinal tract has resulted in a limited number of studies analyzing the microbial composition of the human small intestine. Several studies have relied on sampling from ileostomy bags [19, 20], but such sampling will not be fully representative of the small-intestine microbiome [21]. More recent studies sample directly from the intact small intestine through an endoscopic procedure and have begun to unravel unique relationships between small-intestine microbes and disease [18, 22–25]. An added challenge when quantifying individual microbial taxa from samples of low total microbial biomass is that it can be difficult to distinguish true small-intestine microbes from contamination (e.g., from the oral cavity while sampling or from reagents during sample processing). Additionally, the wide range of total microbial loads in the small intestine across individuals highlights the value of using absolute rather than relative microbial loads when investigating potential associations between small-intestine microbes and physiological factors [9, 26, 27].

In this study, we selected a cohort of 250 individuals from the REIMAGINE study [3] to assess the absolute microbial loads in the human duodenum and their potential relationship with factors related to health and disease. We also surveyed the oral microbiome in a subset of 21 individuals from this cohort to understand the relationship between microbial taxa at these two body sites. We utilized our recently developed digital PCR anchored 16S rRNA gene amplicon sequencing method to provide absolute taxon abundances and filter out contaminants in samples with low microbial abundance [9]. We also used our optimized sample-collection procedure with a custom double lumen sterile closed catheter system and optimized processing steps to minimize oral, gastric and dead microbial contamination [28]. We hypothesized that by capturing the absolute microbial abundances of the human duodenal and oral microbiome we would be able to better understand the makeup of the human duodenal microbiome, improve the understanding of the underlying community structure of SIBO, and determine how microbial load and composition correlate with upper GI symptoms.

## Results

We studied the microbiome of the duodenum and its potential relationship with health and disease in a cohort of 250 patients enrolled in the REIMAGINE study at Cedars-Sinai Medical Center. All patients undergoing esophagogastroduodenoscopy (EGD) without colonoscopy preparation as standard of care were eligible to enroll, resulting in patients with a wide range of GI conditions. We grouped the reason for endoscopy into 11 broad categories (Table S1). The most common (45% of the patient population) reasons for endoscopy were to rule out cancer/polyps and GERD/dyspepsia workup. No healthy controls are currently approved to be included in the study due to the risks associated with the EGD procedure. Summary statistics for patient demographic data and selected metadata categories from the enrollment questionnaire are included in Table S1.

### Total microbial load of the duodenum across patients with GI symptoms is log-normally distributed

**Fig. 1:**
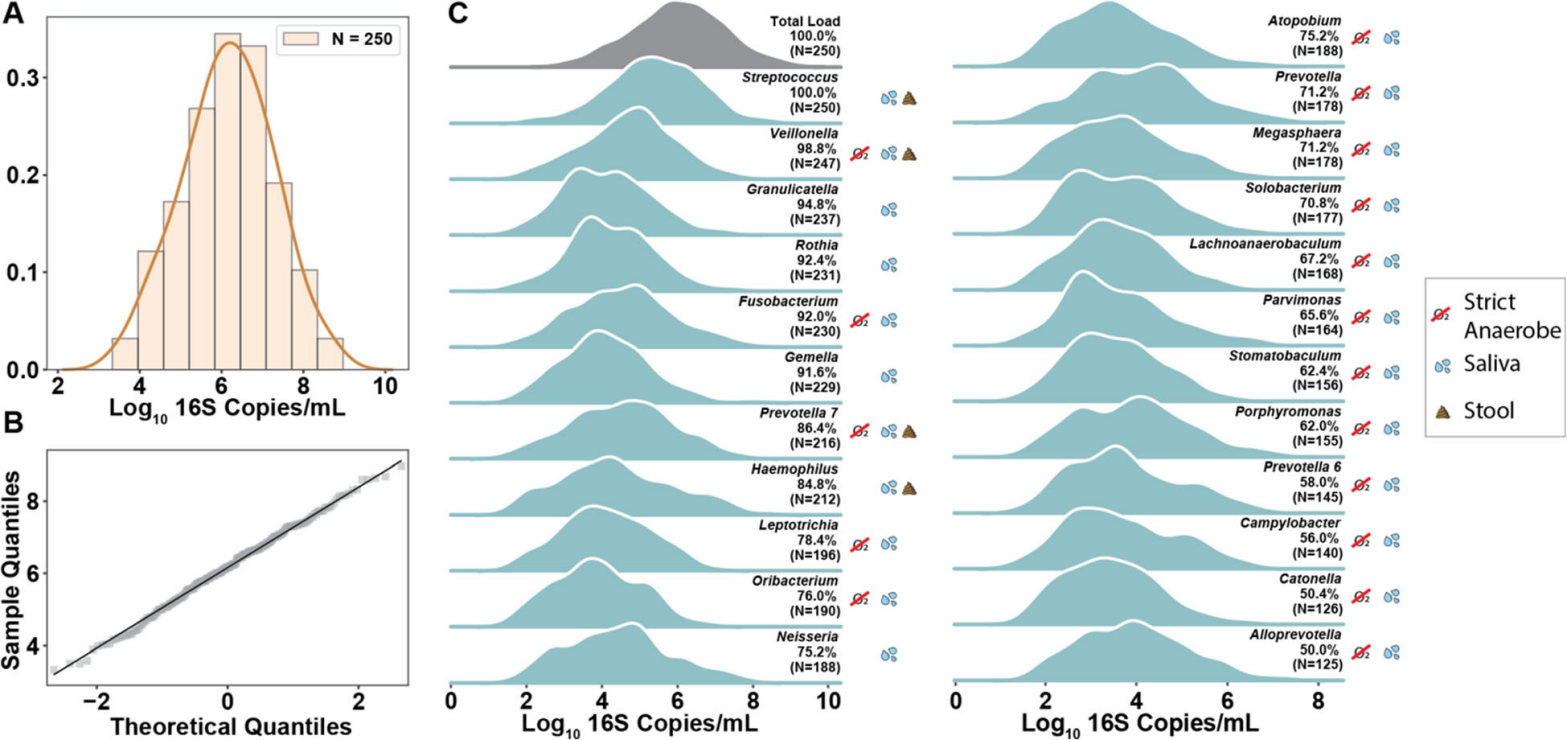
Microbial load distribution across 250 human duodenal aspirate samples. **A** Histogram of the total microbial load in 250 duodenal aspirate samples overlaid with a kernel-density estimate. **B** Quantile-quantile plot comparing the sample distribution of the log_10_-transformed total microbial load in duodenal aspirate samples to a normal distribution. **C** Kernel-density estimate plots showing the absolute abundance distribution for the taxa with greater than 50% prevalence in duodenal aspirates. Prevalence (defined as a taxon’s frequency of occurrence in our dataset) and number of samples with each genus are labeled next to the distribution. A legend indicates strict anaerobes (red line through O_2_) and the location each genus is commonly found (saliva and/or stool) [30, 31]. Classification of taxa as common in stool or saliva was determined by prevalence of ≥ 50% (stool data are not included in this study) in the 16 participants for whom we had paired samples

A digital PCR-based determination of total microbial load [9, 29] from 250 human duodenal aspirates revealed samples that spanned loads from our detection limit of ~ 5 × 10^3^ rRNA gene copies/mL up to nearly 10^9^ copies/mL. The overall distribution of total loads was log-normal with mean = 6.13 Log_10_ copies/mL and standard deviation = 1.12 Log_10_ copies/mL (Fig. 1A, B). A quantile-quantile (QQ) plot was constructed to compare the sample distribution to a log-normal distribution (Fig. 1B). Data from our samples aligning with the *y* = *x* line on a QQ-plot indicate a high similarity between the sample distribution and a theoretical log-normal distribution [32]. Neither age nor gender significantly correlated with total microbial load (Fig. S1). Total microbial load also did not correlate with patient reported intake of probiotics supplements or yogurts, smoking, or usage of proton pump inhibitors (Fig. S2, Table S2). Current antibiotic usage appeared to lower the average total microbial load, but antibiotic usage in the previous 6 months had no impact (Fig. S2, Table S2).

Digital PCR anchored 16S rRNA gene amplicon sequencing [9] (hereafter quantitative sequencing) provided absolute taxon abundances in each sample and a statistical framework for differentiation between real and contaminant taxa (Methods). We first compared the culture counts from aerobic (MacConkey agar) and anaerobic (blood agar) plates to the total load of microbes expected to grow on these plates (Fig. S3). For aerobic plating, we observed a bimodal distribution of combined *Escherichia-Shigella*, *Enterobacteriaceae*, *Enterococcus*, and *Aeromonas* bacterial load from quantitative sequencing and culture and a high correlation between the two measurements (Spearman, 0.61, *P* < 0.001, *N* = 244). For anaerobic plating, we observed lower concordance (Spearman, 0.35, *P* < 0.001, *N* = 244) between quantitative sequencing and culture. This discrepancy could reflect the difficulty in culturing many intestinal microbes [33], especially anaerobes that are initially collected and processed in aerobic environments.

Next, we analyzed the log-transformed absolute-abundance distributions for the most prevalent genera in our dataset (Fig. 1C). We define prevalence as a taxon’s frequency of occurrence in our dataset. *Streptococcus* was present in all 250 samples and followed an approximately log-normal distribution with a mean load that was half an order of magnitude below that of the mean total microbial load and an equal standard deviation. Other genera showed wide-ranging distributions that deviated from normality. For example, *Porphyromonas* appears bimodal with two local maxima whereas *Haemophilus* exhibits a long tail towards higher microbial loads. The 23 most prevalent genera in this study are also commonly found in the oral microbiota [30]. A subset of these genera (*Streptococcus*, *Veillonella*, *Prevotella 7*, *Haemophilus*) are also commonly found in stool samples, indicating possible survival of these genera throughout the entire GI tract [31]. The majority of prevalent genera are either strict or facultative anaerobes, indicating that parts of the duodenal environment are likely anoxic in this patient population.

### Direct transmission of microbes from saliva to duodenum

To investigate whether many of the taxa found in the duodenum originated from the oral cavity we analyzed a subset of 21 patients for whom we had paired saliva and duodenum samples that were collected during the same hospital visit. Digital PCR revealed that the total microbial load in saliva was roughly 2.5 orders of magnitude higher than the total load in the duodenum (Kruskal-Wallis, *P* < 0.001).

**Fig. 2:**
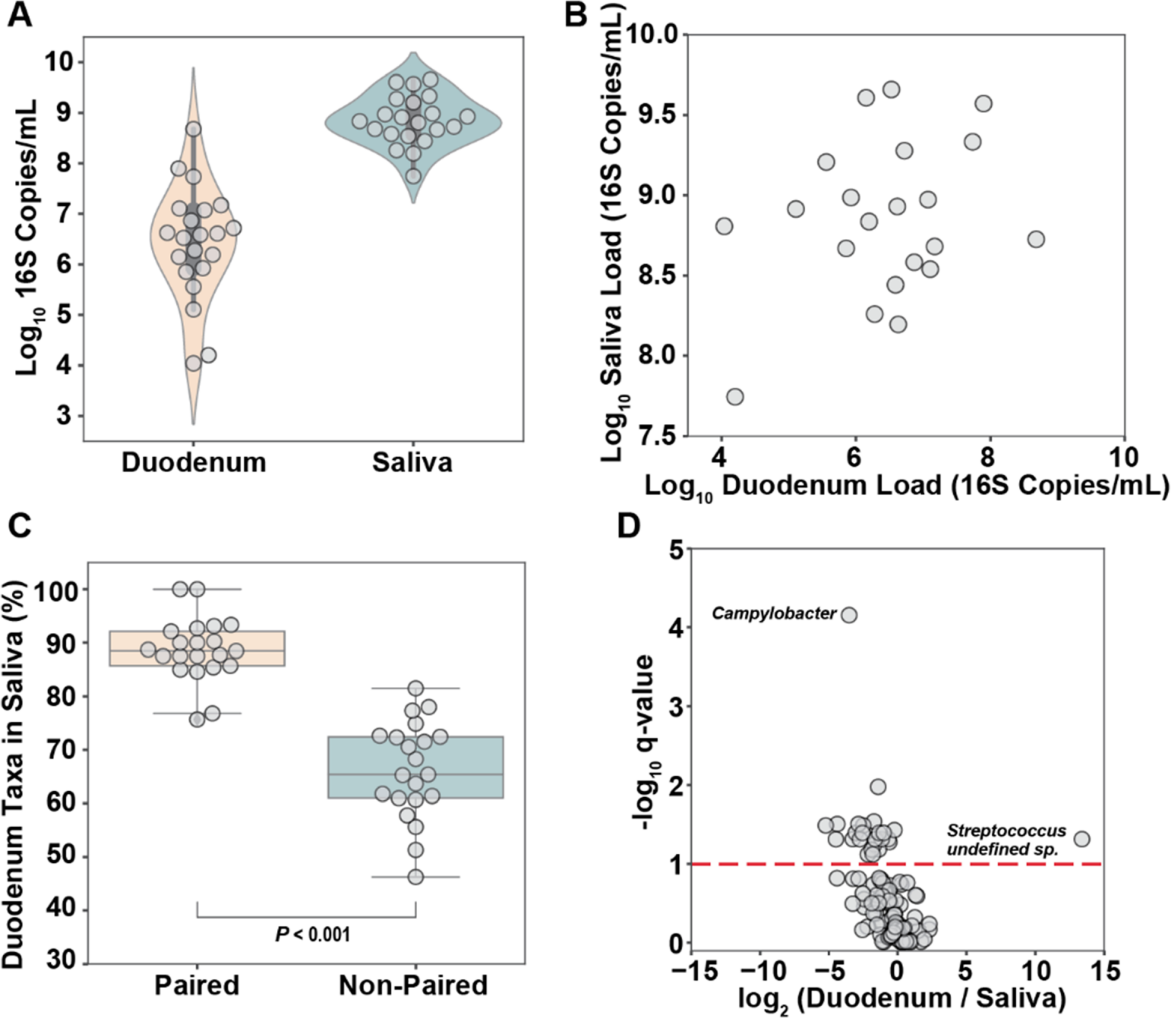
Relationship between saliva and duodenal aspirate microbiomes. **A** Total microbial load of 21 paired duodenal aspirate and saliva samples. **B** No significant correlation between the total microbial load of 21 paired duodenal aspirate and saliva samples. **C** Percentage of taxa in duodenal aspirate samples also present in paired (same patient) vs the average of all non-paired saliva samples (Kruskal-Wallis, *P* < 0.001). **D** Volcano plot showing the ratio of relative abundances of species in duodenum vs saliva samples. The red dashed line indicates a significance threshold at *q* = 0.1 (Kruskal-Wallis with Benjamini-Hochberg correction). Undefined *Streptococcus sp*. classified as *S. pneumoniae* with 80% confidence and one base pair mismatch to common *Streptococcus* taxon found in all samples

Further, the range in saliva total loads was 3 orders of magnitude smaller than the range in total loads of the duodenum samples (Fig. 2A). No significant correlation was observed between the total microbial loads in paired saliva and duodenum samples (Fig. 2B). In this study, all samples were collected with a custom double-sheathed catheter via endoscope (see “Methods” section) that moves beyond the outer sheath before aspirating duodenal fluid. This custom catheter should limit oral microbiota contamination of the duodenum during the procedure. Additionally, the optimized sample-processing protocol (see “Methods” section) should eliminate extracellular DNA from swallowed dead bacteria.

To evaluate the direct transmission of microbes from saliva to duodenum, we compared the shared taxa between paired (same patient) and randomly paired samples from the same dataset. On average, 89% (± 6% S.D.) of the taxa in the duodenum were also found in the paired saliva sample, whereas only 66% (± 9% S.D.) were found in the average of all non-paired comparisons (Fig. 2C, Kruskal-Wallis, *P* < 0.001), suggesting direct transmission of oral taxa to the duodenum. We then looked for genera that were proportionally enriched in either saliva or duodenum samples. *Campylobacter* was present in 21/21 saliva samples but only 10/21 duodenum samples. The absence of *Campylobacter* in about half of the paired duodenum samples indicates the oral cavity may be the preferred niche of *Campylobacter* or that *Campylobacter* has a high sensitivity to the antibacterial properties of the stomach and small intestine [34] (Fig. 2D). In contrast, an undefined species of *Streptococcus* was only found in duodenum samples (6/21) (Fig. 2D). A breakdown of the difference between duodenal and saliva abundance of all taxa is provided in Table S3. These differences in the relative abundance of specific taxa of microbes between paired saliva and duodenum samples also provide evidence against oral contamination in the duodenal samples.

### Taxa co-correlations reveal disruptor taxa

**Fig. 3:**
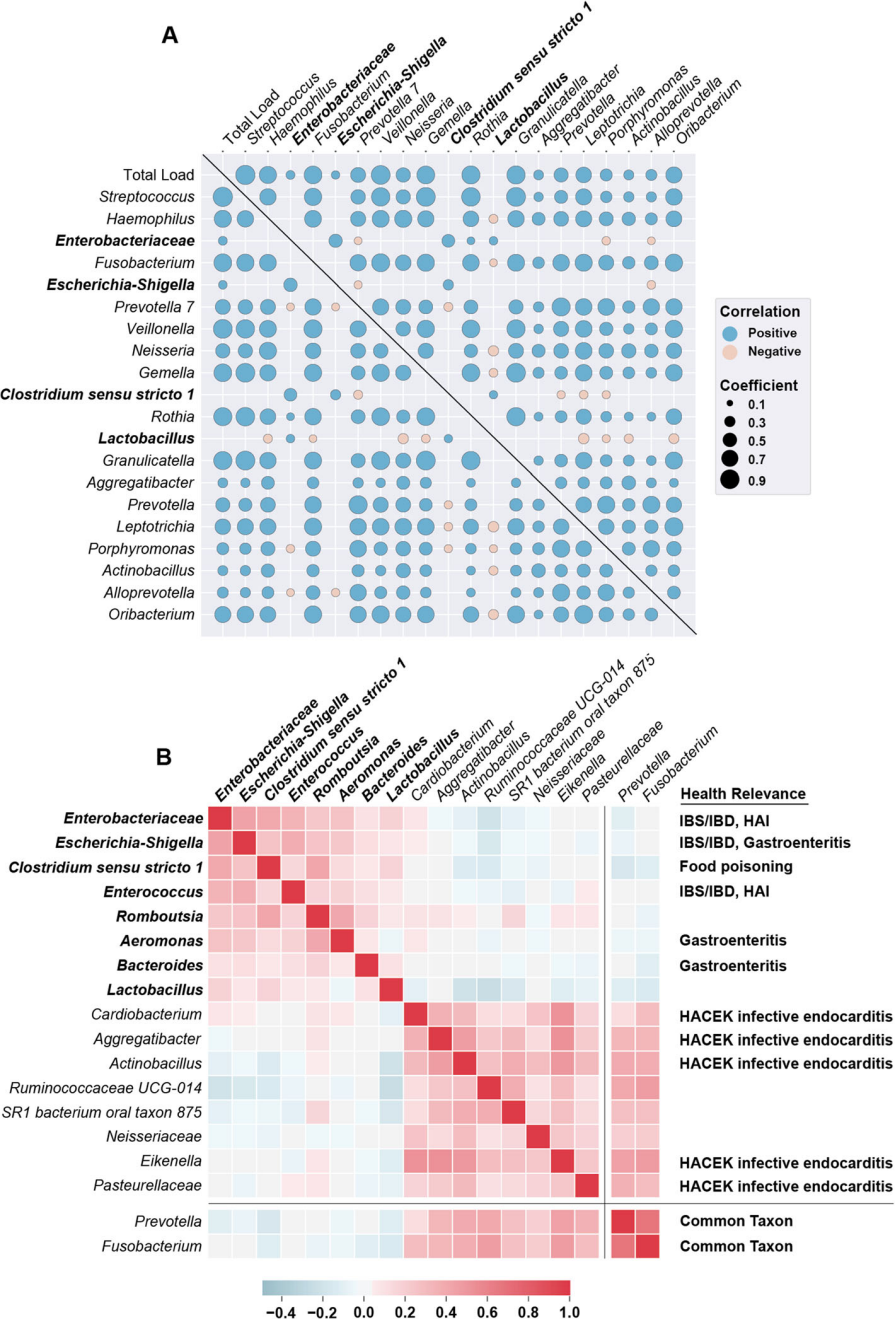
Co-correlations reveal which taxa co-occur in high abundance and which can be considered disruptor taxa. **A** Co-correlation matrix of the top 20 most abundant genera and total microbial load. Only significant correlations (*q* < 0.1, Benjamini–Hochberg correction) are shown. Color of each marker is determined by the sign of the Spearman’s correlation coefficient and size of each marker is determined by the magnitude of the coefficient. Disruptor taxa labels are bolded. **B** Clustered co-correlation matrix of the top 16 genera ranked by the difference between their maximum abundance and mean abundance. Two common genera in the dataset are shown at the bottom for reference. The color of each square indicates the Spearman correlation coefficient from negative (blue) to positive (red). Disruptor taxa labels are bolded. Taxa with known relevance to human health are indicated. *Enterobacteriaceae* and *Escherichia-Shigella* are unique sequence variants from the *Enterobacteriaceae* family but only *Escherichia-Shigella* could be classified at the genus level. HAI=hospital acquired infection; IBS, irritable bowel syndrome; IBD, inflammatory bowel disease; HACEK, *Haemophilus*, *Aggregatibacter*, *Cardiobacterium*, *Eikenella*, *Kingella*

We assumed that the taxa with the highest absolute abundance would have the highest potential for impacting the host. Thus, we began by analyzing the relationships between the top 20 most abundant genera. A co-correlation heatmap of these taxa revealed several distinct motifs (Fig. 3A): (1) taxa whose absolute loads had a high correlation with total load, (2) taxa whose absolute loads had a higher co-correlation with another taxon’s absolute load than with total microbial load, (3) taxa with a mutually exclusive relationship with almost all other abundant taxa. Examples of the first motif are in the first column/row of the co-correlation heatmap in Fig. 3A. Correlation with total load was often an indicator of a prevalent taxon because the variance in total microbial load was larger than the variance in relative abundance. When two taxa have a higher co-correlation with each other than with total load (motif 2), it potentially indicates these taxa share preferred environmental factors or directly cooperate. One group of these co-correlating taxa that included several *Prevotella* species and a species of *Porphyromonas* matches a known shared metabolic niche in the oral cavity [35, 36] (Table S4).

Several genera stood out as having no significant correlation with almost all other abundant taxa (motif 3): *Enterobacteriaceae*, *Escherichia-Shigella*, *Clostridium sensu stricto 1*, and *Lactobacillus* (Fig. 3A). For clarification, throughout the manuscript our references to *Enterobacteriaceae* and *Escherichia-Shigella* refer to unique sequence variants from the *Enterobacteriaceae* family, but only *Escherichia-Shigella* could be classified at the genus level. Based on evidence from a previous study [18] using the REIMAGINE cohort that found *Klebsiella* in several samples, we decided to measure the abundance of *Klebsiella* via qPCR in all samples containing a high abundance (at least 10^5^ 16S rRNA gene copies/mL) of *Enterobacteriaceae*. We found that the majority (16/22) of the samples with a high abundance of *Enterobacteriaceae* contained *Klebsiella* (Fig. S4A)*.* Furthermore, in the samples containing *Klebsiella,* there was a high correlation (Pearson, 0.88, *P* < 0.001) between *Klebsiella* load and *Enterobacteriaceae* load (Fig. S4B). These taxa appeared to disrupt the commonly observed microbial structure (i.e., the prevalent taxa that generally co-correlate with one another) of the duodenal microbiome. This pattern of mutual exclusivity can be represented algorithmically by sorting all taxa by the difference between their maximum abundance and their mean abundance. Practically, this means that these disruptors are relatively rare (i.e., present in a small fraction of samples), but when they are present they usually dominate, excluding other common taxa. A clustered heatmap of the top 16 taxa as ranked by the difference in their maximum and mean abundances reveals two taxonomic signatures (Fig. 3B). The first signature in the top left of the heatmap contained the mutually-exclusive taxa from the co-correlation heatmap, along with *Enterococcus*, *Romboutsia*, *Aeromonas*, and *Bacteroides*. The second signature contained taxa that were generally found in lower abundance, many of which are from the HACEK (*Haemophilus*, *Aggregatibacter*, *Cardiobacterium*, *Eikenella*, *Kingella*) group of organisms associated with infective endocarditis [34]. However, the second group also clustered with more common taxa in this dataset, such as *Prevotella* and *Fusobacterium*. Thus, we initially labelled all eight of the taxa in the first taxonomic signature as “disruptors” (Fig. 3B, bolded taxa) because their presence appeared to be mutually exclusive with many other common taxa.

### Aerobic disruptor taxa displace strict anaerobes and decrease diversity

**Fig. 4:**
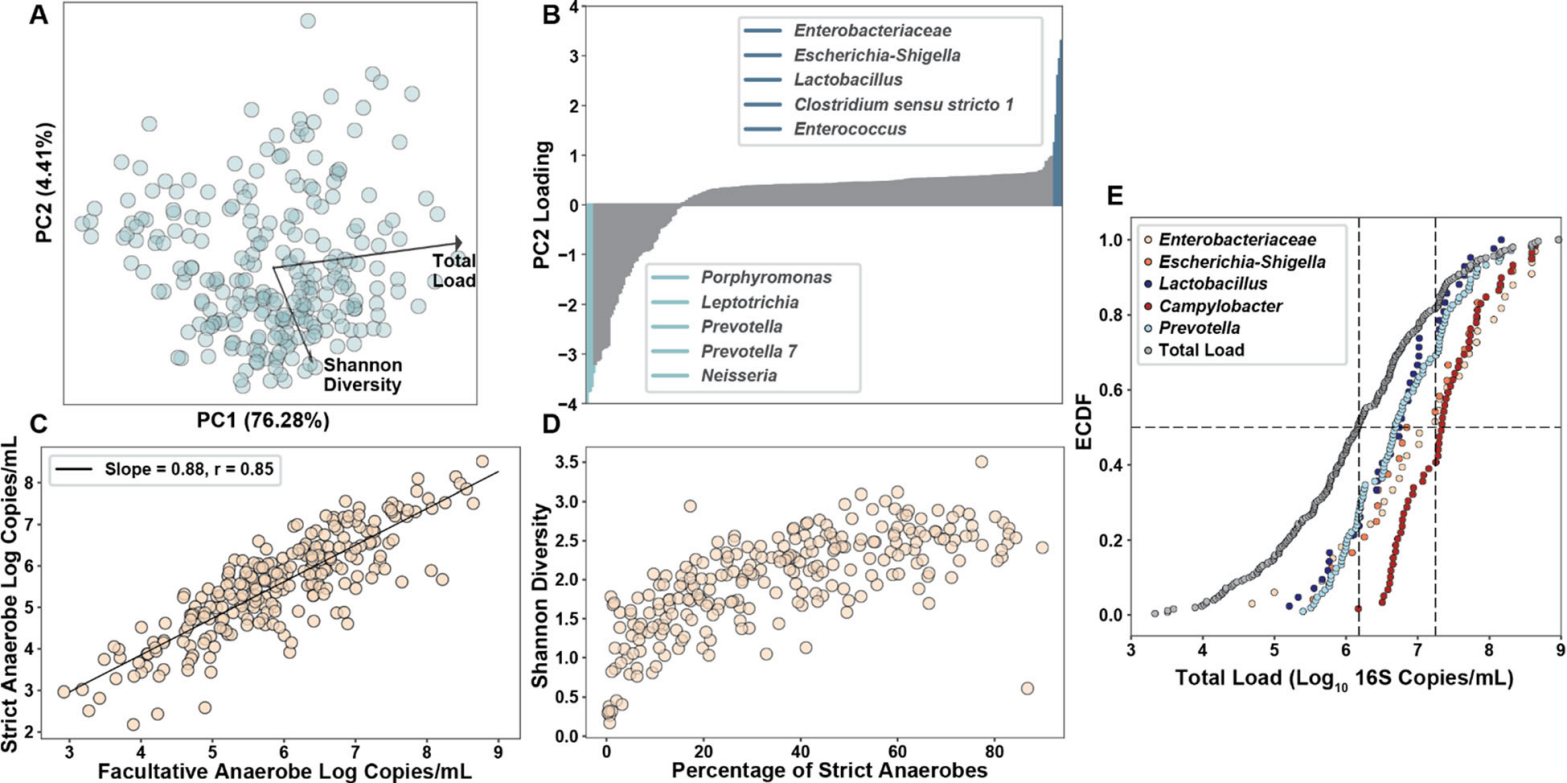
Strict anaerobes and disruptor taxa control diversity. **A** PCA plot of absolute microbial abundances at the genus level with the top two correlated metadata variables overlaid. **B** Feature loadings for principal component 2. Top five value-ranked genera in each direction (positive and negative) are highlighted and labeled. **C** Correlation between the strict anaerobic microbial load and facultative anaerobic microbial load. **D** Relationship between the percentage abundance of strict anaerobes and Shannon diversity index. **E** Empiric cumulative distribution function (ECDF) plot for *Enterobacteriaceae* (*N* = 33), *Escherichia-Shigella* (*N* = 24), *Campylobacter* (*N* = 59), *Lactobacillus* (*N* = 42), and the common taxa *Prevotella* (*N* = 104)

After performing the co-correlation analysis, we ran a principal component analysis (PCA) on the absolute taxon abundances to investigate the drivers of variance in the dataset (Fig. 4A). Total loads spanned 5 orders of magnitude, accounting for most of the variance. Total load cleanly separated samples along the PC1 axis. The second most explanatory axis, PC2, strongly correlated with the Shannon diversity index of samples (Spearman, 0.74, *P* < 0.001, *N* = 250). Ranked feature loadings for PC2 (Fig. 4B) indicated that many of the disruptor taxa (dark blue) are the main drivers of separation in the positive direction of PC2 whereas the five taxa driving most of the separation in the negative direction (light blue) of PC2 consisted of four strict anaerobes (*Porphyromonas*, *Leptotrichia*, *Prevotella*, *Prevotella 7*) and one obligate aerobe (*Neisseria*). It should be noted that many more taxa were strongly associated with the negative direction of PC2 than the positive direction. This separation matches well with the mutual exclusivity seen between the disruptor taxa and other organisms in the co-correlation analysis. The two disruptor taxa with the highest loads are aerobic pathogens from the *Enterobacteriaceae* family and the taxa most associated with the negative direction of PC2 were strict anaerobes, so we next took a closer look at the composition of strict vs facultative anaerobes in each sample. We found a nearly 1:1 correlation between the strict and facultative anaerobe loads across all samples (Fig. 4C). Additionally, the fraction of strict anaerobes in a sample was strongly correlated (Pearson, 0.71, *P < 0.001*, *N* = 250) with Shannon diversity (Fig. 4D), indicating that the disruptor taxa appear to be mutually exclusive with strict anaerobes and the “bloom” of absolute abundance of disruptors decreases Shannon diversity. Furthermore, in half of the samples containing the two most common disruptor taxa (*Enterobacteriaceae* and *Escherichia-Shigella*), the total microbial loads were greater than 10^7^ 16S rRNA gene copies/mL, indicating a clear enrichment of disruptor taxa in samples with higher than average total microbial loads (Fig. 4E). This signature of higher than average total microbial loads and mutual exclusivity with other microbes has been observed in some pathogenic microbial species [37, 38].

### Absolute load of disruptor taxa correlates with SIBO and GI symptoms

**Fig. 5:**
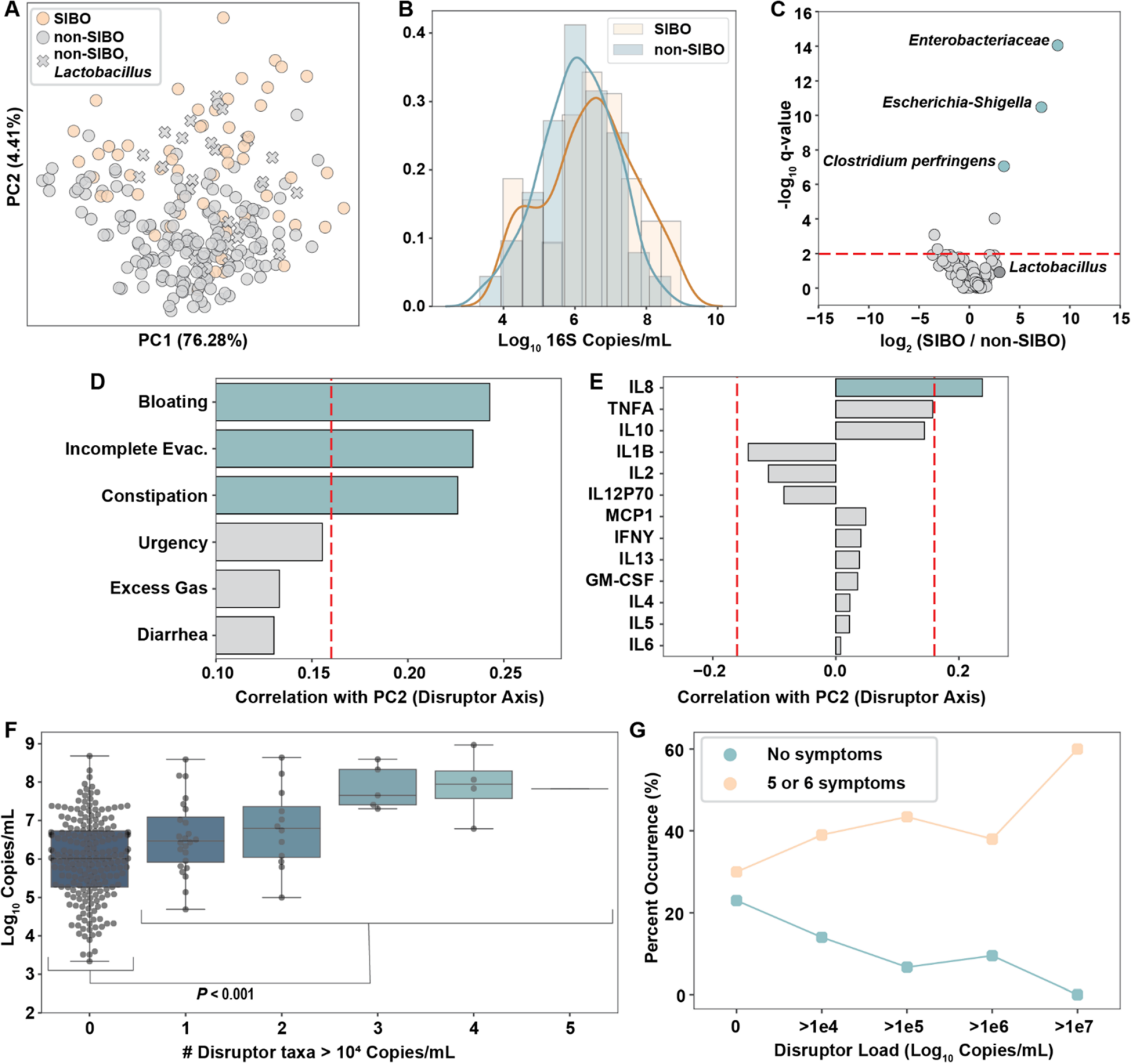
Disruptor species are dominant in SIBO samples and correlate with GI symptoms and the inflammatory cytokine IL8**. A** Principal component analysis (PCA) of absolute microbial abundances at the genus level. Colors indicate non-SIBO (grey) or SIBO (orange) participants as determined by culture. “X” markers indicate samples from non-SIBO participants that contained *Lactobacillus*. The PC1 axis correlates with total load and the PC2 axis correlates with the abundance of disruptor taxa. **B** Histogram with overlaid kernel-density estimate of the total microbial loads in samples from SIBO and non-SIBO participants. **C** Volcano plot indicating the taxa that differed between SIBO and non-SIBO samples. The red dashed line indicates the significance threshold at *q* = 0.01. **D** Correlation between PC2 (disruptor axis) and patient-reported symptom scores (on a 0–100 scale). The red dashed line represents significance threshold at *q* = 0.05. **E** Correlation between PC2 and patient serum cytokine levels. The red dashed lines represent the significance thresholds at *q* = 0.05. **F** Boxplot indicating increasing average total microbial load with increasing number of disruptor taxa with loads greater than 10^4^ rRNA gene copies/mL (not including *Lactobacillus*). A significant difference between total load in samples with zero disruptor taxa and total load in samples with at least 1 disruptor taxa was observed (*P* < 0.001). **G** Percentage of samples from patients with either 0 symptoms or 5–6 symptoms (out of 6 categories) for individuals with varying loads of disruptor taxa (not including *Lactobacillus*)

To determine whether disruptor taxa are associated with disease or GI symptoms we began by looking at patients with and without SIBO (SIBO classification was made based on aerobic culture results, ≥ 10^3^ CFU/mL of duodenal aspirate [10]). Coloring the PCA plot by SIBO classification indicates a clear enrichment of patients with SIBO in the positive direction of the disruptor taxa axis (Fig. 5A). We observed slightly but not significantly higher total microbial loads in samples from patients with SIBO vs without SIBO (Fig. 5B). However, comparing the absolute abundance of specific taxa between the SIBO and non-SIBO samples by Kruskal-Wallis showed that the three taxa whose abundances differed the most between SIBO and non-SIBO (*Enterobacteriaceae*, *Escherichia-Shigella*, and a *Clostridium* which, based on the V4 region of the 16S rRNA gene, was classified as *Clostridium perfringens*) were also the three most common disruptor taxa in all samples (Fig. 5C). This enrichment of disruptor taxa, but not total microbial load, in SIBO samples indicates that overgrowth of specific taxa drives the current clinical classification of SIBO. Additionally, using disruptor taxa load as the criterion for SIBO classification agreed well (80%) with the classification by the gold-standard method, aerobic aspirate culture (Fig. S5). *Lactobacillus* abundance was similar in SIBO and non-SIBO samples (Fig. 5C) even though it co-correlated with many of the disruptor taxa (Fig. 3B). Most of the non-SIBO samples that clustered with SIBO samples on the upper part of the PC plot contained *Lactobacillus* (Fig. 5A). *Lactobacillus* does not grow on the aerobic (MacConkey agar) plates used for SIBO classification, which could explain why these samples cluster together by sequencing but are not classified as SIBO by culture.

Patient-reported GI symptom scores (on a 0–100 scale) were correlated with the disruptor taxa axis (PC2). Bloating, incomplete evacuation, and constipation had the highest correlation with the disruptor taxa axis, whereas correlations between urgency, excess gas, or diarrhea and the disruptor taxa axis were much weaker (Fig. 5D). There was a weak positive correlation between the disruptor taxa axis and serum interleukin 8 (IL8) levels (Spearman, 0.24, *P* < 0.001, *N* = 232), indicating a potential neutrophil-related response (Fig. 5E). However, none of the symptoms or cytokines had a significant correlation with the total load axis (PC1). One taxon, which based on the V4 region of the 16S rRNA gene was classified as *C. perfringens,* was the only one that, when present in patients, coincided with a significant increase (Kruskal-Wallis, *P = 0.039*) in serum IL8 levels (Fig. S6). However, there were only 9/250 samples with *C. perfringens,* limiting our ability to draw conclusions about this relationship. Although the two disruptor taxa with the highest absolute abundance (*Enterobacteriaceae* and *Escherichia-Shigella*) were enriched in high total microbial load samples, *Lactobacillus* did not follow this trend. *Lactobacillus* was found in samples with total microbial loads that were similar to the total loads of samples containing common taxa like *Prevotella* (Fig. 4E). Additionally, in patients with high disruptor taxa loads (after excluding *Lactobacillus* load) the presence of *Lactobacillus* at greater than 5 × 10^4^ copies/mL negatively correlated with bloating symptoms (Fig. S7). These two facts led us to believe *Lactobacillus* likely has a more nuanced relationship with the host than the other taxa we classified as disruptors. Thus, we removed *Lactobacillus* from our list of disruptor taxa in our analyses of the association of disruptors with total load (Fig. 5F) and GI symptoms (Fig. 5G). When multiple disruptor taxa were present, there was a significant increase in total microbial load (Kruskal-Wallis, *P* < 0.001; Fig. 5F).

Patient-reported symptom scores are inherently qualitative, so to test whether disruptor taxa loads were correlated with more severe GI symptoms, we turned the 0-100 scores into a binary yes/no variable, representing a severe symptom, by drawing a threshold at the median score reported for each symptom (Fig. S8). We then calculated the percentage of patients with zero severe symptoms and the percentage of patients with many severe symptoms (people reporting severe symptoms in 5–6 of the 6 symptom categories) as a function of disruptor taxa loads (Fig. 5G). We made three observations. First, at higher disruptor loads, patients were more likely to have more severe GI symptoms. Second, none of the patients with disruptor loads greater than 10^7^ copies/mL (*N* = 10) had zero symptoms whereas 60% of them had 5 or 6 symptoms. Of the patients without disruptor taxa (*N* = 153), 23% had zero symptoms and 30% had 5 or 6 symptoms. Disruptor loads may also be higher as a function of age, all but one of the individuals with disruptor loads greater than 10^6^ copies/mL (*N* = 23) were older than 50 (Fig. S9). The absolute and relative abundances of disruptor taxa did not correlate (Fig. S10), preventing the clear connection between abundant symptoms and high absolute loads of disruptor taxa from being observed when analyzing only relative abundances.

## Discussion

In this study, we utilized quantitative sequencing to determine the total and taxon-specific loads from the duodenum of 250 patients undergoing EGD as standard of care. We showed that the total microbial load in the duodenum of these patients spans 5 orders of magnitude and follows a log-normal distribution. Paired saliva-duodenum samples revealed that on average 89% of the taxa in the duodenum were also present in paired saliva samples, suggesting potential transmission of taxa from the oral cavity. Co-correlation analysis of the most abundant taxa revealed a distinct taxonomic motif of “disruptor” taxa that, when present, dominate over other taxa. The most common of these disruptor taxa were aerobic pathogens from the *Enterobacteriaceae* family and were negatively correlated with the presence of strict anaerobes and diversity. In addition to the apparent community disruption, disruptor taxa were enriched in many patients classified as having SIBO and high loads of disruptors correlated with a high prevalence of severe GI symptoms.

### Human vs mouse small-intestine microbiome

Several findings from this study emphasize how different the small-intestine microbiome is between mice and humans. Our previous study revealed that the coprophagic nature of mice resulted in total microbial loads spanning approximately one order of magnitude from 5 × 10^8^–5 × 10^9^ 16S rRNA gene copies/mL [8] in the small intestine while our human cohort spanned 5 orders of magnitude with a median of 10^6^ copies/mL (Fig. S11). Additionally, neither the most common disruptor family, *Enterobacteriaceae*, nor any of the taxa with at least 50% prevalence in this study were commonly found in our previous study examining microbial loads in the mouse small intestine [8]. Instead, in that study we found that the mouse small intestine was dominated by *Lactobacillus* and, as a result of coprophagy, several stool microbes [8]. The total microbial load of stool is similar between mice and humans [39] and they both share several common taxonomic groups [40]. These differences should be considered when using mice to model human health or disease impacted by the small intestine.

### Value of quantitative analysis

The nearly 5 orders of magnitude spread in total microbial loads in the duodenum of these patients revealed the value of utilizing an absolute abundance measurement technique when analyzing microbial communities. Analyzing absolute abundances of individual taxa also let us filter out likely contaminants using Poisson loading statistics, which is critical for samples with low microbial abundance, such as those sometimes found in the human small intestine [41, 42]. The range of total loads in saliva and in stool each appear to be smaller than in the duodenum, closer to two orders of magnitude, which likely relates to differences in residence times, nutrient availability, and host defenses at these two sites compared with the small intestine [39]. Another benefit of using absolute rather than relative abundance measurements is the improved accuracy of correlations between microbes and host phenotype. For example, the 10 patient samples with the greatest disruptor loads had the highest prevalence of severe GI symptoms, but these samples had relative abundances of disruptor taxa that ranged from 8 to 97%. This wide range of relative abundances made samples with high disruptor loads indistinguishable from samples with intermediate disruptor loads when analyzing relative abundances.

### Microbial connection between oral cavity and small intestine

The majority (89%) of identified microbial taxa in the paired duodenum samples were also present in the paired saliva samples. Our data supports the hypothesis of oral-duodenal transmission of microbes but a larger paired study utilizing shotgun metagenomic sequencing techniques would provide stronger evidence for this claim. Survival of microbes after ingestion is likely dependent on many host factors, including stomach-acid levels, bile secretions, antimicrobial-peptide production, and GI motility. The bimodal taxon abundance distributions (Fig. 1C) observed for some taxa, including *Prevotella*, may indicate two subsets of patients with distinct stomach and/or duodenal environments that allow for differential abundance of specific taxa. For example, *Campylobacter concisus*, one of the most common oral *Campylobacter* species, is known to be sensitive to both stomach and bile acids [34]. Therefore, one could hypothesize that if a patient had low levels of stomach or bile acids some *C. concisus* may survive ingestion. Low-acid conditions could also allow many other bacteria to survive transit to the duodenum, resulting in higher total microbial loads in the small intestine. We suspect we observed something similar in our samples; the *Campylobacter* genus was only found in samples with greater than average total microbial loads (Fig. 4E). However, we did not observe a relationship between total microbial loads in the duodenum and the patients’ use of proton pump inhibitors (PPI), which are known to reduce acid production. PPI impact on survival of microbes between the oral cavity and duodenum may be dependent on how recently the PPI was taken, however this information was not collected from patients in the REIMAGINE study. A conclusive comparison of the relative importance of various factors affecting bacterial survival in the duodenum would require additional information on small-intestine secretions of bile acids and antimicrobial peptides in these patients.

Several common oral microbes have been implicated in GI diseases when present in stool [30, 43]. A high microbial load in the small intestine could increase the likelihood of these microbes surviving all the way down the GI tract. The shared taxa between the small intestine and oral microbiota in our paired saliva-duodenum samples provides evidence that blooms of opportunistic pathogens in the mouth could also lead to colonization in the SI [30]. In this study, only 1 of the 21 paired duodenum-saliva samples contained disruptor taxa in the duodenum, but these taxa were not present in the corresponding saliva sample. Several *Enterobacteriaceae* species have been identified in oral samples [44] but usually at a low frequency in healthy populations. Many *Enterobacteriaceae* species are introduced into the gut from contaminated food and water sources [45] which would likely result in only transient oral residence. However, persistent oral *Enterobacteriaceae* species have been linked to the use of dentures and the presence of periodontal disease [46]. All the taxa we classified as disruptors in this study are more frequently found in stool than in the small intestine or oral cavity [30, 31]. Further studies should be performed to determine the source of disruptor taxa in the upper GI tract.

A number of taxonomic groups we identified in the duodenum have members known to be opportunistic pathogens. Beyond disruptor taxa, several taxa from the HACEK group of organisms [47] associated with infective endocarditis were found in high abundance in the duodenum. The route that these and other opportunistic pathogens take to reach the blood stream is not clear but our data show that the HACEK organisms are not limited to the oral cavity. The same traits that allow them to colonize the mouth and heart (biofilm production [48], and general resistance to most host secretions) likely contribute to their ability to survive in the small intestine. Additionally, in mouse models, the transmission of opportunistic pathogens, like *Klebsiella*, from the oral cavity to the intestine has been shown to induce inflammation [30]. The oral cavity presents a potential reservoir for a wide range of opportunistic pathogens that have been linked to GI disorders.

### Potential relationship between oxygen and disruptor taxa

Several colonic GI disorders are linked to increased oxygen levels in the lumen resulting from decreased epithelial integrity and inflammation [49]. However, the barrier properties of the small intestine, an absorptive organ, are different from those of the colon. To our knowledge, shifts in absolute abundance of microbes capable of aerobic respiration and anaerobes have not been quantitatively studied previously in the human small intestine. The highly correlated abundance of both strict and facultative anaerobes that we observed could be a function of the oxygen gradients in the gut from the epithelial surface to the center of the lumen [50]. In our study, when diversity collapsed and disruptor taxa bloomed, the microbial composition shifted away from strict anaerobes to taxa capable of aerobic respiration. One clear outlier was a *Clostridium* classified as *C .perfringens,* which is a strict anaerobe but was highly correlated with the *Enterobacteriaceae* genera classified as disruptors. Previous mutualistic relationships between aerobic and anaerobic species that could help facilitate colonization have been observed in other studies with *Bacteroides fragilis* and either *Klebsiella pneumonia* or *Escherichia coli* [51, 52]. We have previously hypothesized that the surprising coexistence of aerobe-anaerobe communities can occur in multi-stable systems, and that these communities can persist due to hysteresis [51]. Although multi-stability and hysteresis have not yet been documented in the gut microbiome, this phenomenon could explain the unexpected coexistence and persistence of aerobe-anaerobe communities in the small intestine.

### Disruptor taxa predict SIBO classification and likelihood of GI symptoms

Clinically, SIBO is classified by culture of duodenal aspirates on aerobic MacConkey agar or measurement of exhaled hydrogen and methane after intake of a fermentable sugar solution [10, 53]. The main disruptor taxa (*Enterobacteriaceae*) grow well on MacConkey agar plates, which may explain the high correlation between SIBO classification and samples with disruptor taxa. It is commonly hypothesized that overgrowth of these taxa in the small intestine is responsible for the gas production detected during a breath test, and our study further supports this understanding because we found a correlation between bloating symptoms (attributable to gas production) and disruptor taxa. Future studies should determine whether individuals with and without high loads of disruptor taxa yield positive breath test results. Our findings support a strong relationship between overgrowth of specific disruptor taxa and GI symptoms in subjects with SIBO. High total microbial load alone in the small intestine was not associated with GI symptoms usually observed in subjects with SIBO and other GI conditions and diseases. Microbial culture is never perfect and will not capture all taxa associated with SIBO and GI conditions. However, our data suggest that SIBO diagnosis via microbial culture should focus on quantification of a specific group of disruptor taxa (*Enterobacteriaceae*) rather than total microbial load. Additionally, SIBO diagnosis via quantitative sequencing should focus on the absolute abundance of the seven disruptor taxa identified in this study.

*Lactobacillus* seemed to be an exception among the disruptor taxa in several ways. It commonly co-occurred with other disruptors; however, it was also present in many “normal” samples at low abundance. Additionally, when present at high total loads in the presence of other disruptor taxa, *Lactobacillus* load had a negative correlation with bloating score. However, *Lactobacillus* also dominated several samples that had no other disruptor taxa but had high symptom scores. It should also be noted that individuals taking probiotics (*N* = 49) did not have increased prevalence or abundance of *Lactobacillus* in the duodenum. Overall, finer taxonomic resolution may be required to decipher the role of different *Lactobacillus* species and strains. Their impact on human health is likely also dependent on the overall microbial community and host environment.

Although most patients in this study have various GI complications that could result in abdominal symptoms independent of a microbial component, patient samples with high loads of disruptor taxa had a substantially higher likelihood of having many severe GI symptoms. However, total microbial load alone did not associate with GI symptoms. Of the 13 cytokines and chemokines measured, only IL8 levels were significantly higher in the serum of patients with disruptor taxa, potentially indicating an associated local inflammatory process. Future studies that analyze biopsy transcriptomes would be needed to determine whether there is an associated host response, such as immune infiltration or epithelial stress responses in regions with disruptor taxa and/or high total microbial loads.

We initiated this study with four expectations, only one of which was supported by our data. Because mice are coprophagic and humans are not, we expected to see a dramatic difference between mouse and human small-intestine microbiomes. We indeed observed large quantitative and qualitative differences between the two. However, we were more surprised and educated by the three expectations that were shown to be incorrect. First, we expected microbial load in the human duodenum to have a bimodal distribution, with low microbial loads for non-SIBO patients and much higher load for SIBO patients, which our findings did not support (Fig. 5B). Second, because stomach acid and bile acid secretions isolate the duodenum from the upper GI tract and because the unidirectional flow of digesta and the ileocecal valve isolate the small intestine from the colon, we expected to find a unique population of microbes in the duodenum. We were surprised by the extent to which the oral microbiota appeared to influence the small-intestine microbiota (Figs. 1C and 2). Third, we expected to see microbiomes dominated by taxa generally thought of as commensals like *Lactobacillus* and *Bifidobacterium*. We were surprised by the prevalence and abundance of taxa known to be human pathogens (Figs. 1C and 3B), especially given that the small intestine is an immune-rich, absorptive organ with a loose mucus structure that likely permits substantial exposure to microbial cells and microbial-associated proinflammatory molecules.

### Limitations

An acknowledged limitation of the study is that there are no healthy controls. All participants had some GI condition warranting the EGD procedure, which could bias our dataset and mask our ability to perceive relationships between microbial abundances and patient symptoms. New sampling techniques may be required to reduce the procedural risk involved with sampling healthy controls. Additionally, all collected samples in this study were from the lumenal contents of the duodenum. Distal regions of the small intestine may reveal further insights, and mucosal biopsies could be more indicative of mucosa-associated microbes that interact closely with the host. Although short amplicon sequencing allowed for more samples to be included in this study, utilizing shotgun sequencing approaches to reveal species- and strain-level resolution could provide additional insights, especially with regard to disruptor taxa and potential transmission of taxa from saliva to the duodenum. Additionally, DNA-based analyses can only inform which microbes are in a sample, not whether they are actively performing a function. RNA-based analyses, either 16S rRNA or meta-transcriptomics, may shed additional light on which microbes are resident vs transient members of the duodenum and what functions they are performing. Finally, to truly unravel the connection between oral-to-small intestine microbial transmission and small-intestine microbe-host interactions, a more extensive characterization of the host is needed. Specifically, studies are needed to establish how variations in stomach acid levels, bile secretions, and GI motility impact the abundance and composition of small-intestine microbiota and in turn how the abundance and composition of small-intestine microbiota impacts immune and epithelial cell responses.

## Conclusions

This study, with its acknowledged limitations, provides the largest dataset of the absolute abundance of microbiota from the human duodenum to date. We show a clear relationship between the human oral microbiota and that of the duodenum. Furthermore, absolute taxon abundances in the duodenum reveal a distinct subset of disruptor taxa, associated with human pathogens, that appear to displace common strict anaerobes. These same disruptor taxa are enriched in some individuals classified with SIBO and the absolute abundance of these disruptor taxa were associated with more severe GI symptoms. Future studies are needed to establish the host factors that control total microbial load in the duodenum, the mechanism of appearance and persistence of disruptor taxa, and how these disruptor taxa interact with the host.

## Methods

### Study population and design

The REIMAGINE (Revealing the Entire Intestinal Microbiota and its Associations with the Genetic, Immunologic, and Neuroendocrine Ecosystem) study was conceived to explore the relationships between the small-intestine microbial populations and different conditions and diseases [3]. Male and female subjects aged 18–80 years undergoing standard-of-care upper endoscopy (esophagogastroduodenoscopy, EGD) without colon preparation were prospectively recruited. All subjects were required to fast (from both solids and liquids, including water) starting at midnight the night before the procedure. The study protocol was approved by the Institutional Review Board (IRB) at Cedars-Sinai Medical Center, and subjects provided written informed consent prior to participation (IRB Protocol: 00035192). Data presented here represents a retrospective analysis of this prospectively collected information.

### Questionnaires

Prior to EGD, all subjects completed a study questionnaire documenting demographic information and family and medical history, including GI disease and bowel symptoms, medication use, use of alcohol and recreational drugs, travel history, and dietary habits and changes. Subjects also reported any known underlying conditions, such as GI diseases and disorders, neurologic disease, hematologic disease, autoimmune disease, kidney disease, heart disease, and cancer. All medical information provided by subjects was verified through audits of medical records. All data were de-identified prior to analysis.

### Blood collection and analysis

After completing the study questionnaire, fasting blood samples were collected in BD Vacutainer SST tubes (Becton Dickson, Franklin Lakes, NJ, USA). Levels of circulating pro- and anti-inflammatory cytokines and chemokines were analyzed on a Luminex FlexMap 3D (Luminex Corp., Austin, TX, USA) using a bead-based multiplex panel that included: GM-CSF, IFNγ, IL10, IL12P70, IL13, IL1B, IL2, IL4, IL5, IL6, IL8, MCP1, and TNFα (EMD Millipore Corp., Billerica, MA, USA, cat. #HCYTOMAG-60K).

### Saliva and small-intestine lumenal sample collection

Prior to EGD procedure, saliva was collected in a sterile 5 mL tube. During the EGD procedure, samples of duodenal lumenal fluid were procured using a custom-designed sterile aspiration double-lumen catheter (Hobbs Medical, Inc.) [28]. Duodenal aspirates (DA) were collected using a custom-designed sterile inner catheter which was pushed through a sterile bone wax cap only after the endoscopist entered the second portion of the duodenum, in order to reduce contamination from the mouth, esophagus, and stomach. After collection, samples were immediately placed on ice and transferred to the laboratory for further analysis.

### Aspirate processing and microbial culture

Prior to microbial culture, an equal volume of sterile 6.5 mM dithiothreitol (DTT) prepared with RNase and DNase PCR-grade sterile water was added at a 1:1 ratio to each saliva and duodenal aspirate (~ 1 mL) and the samples were vortexed until fully liquified (~ 30 s) as described previously [28]. A 100-μl aliquot of each duodenal sample (DA + DTT) was then serially diluted with 900 μL sterile 1× PBS and plated on MacConkey agar (Becton Dickinson), and on blood agar (Becton Dickinson). Plates were incubated at 37 °C for 16–18 h under aerobic (MacConkey) or anaerobic (blood agar) conditions. Plates without bacterial growth after 18 h were re-incubated for an additional 18 h. Colony forming units (CFU) were then counted electronically using a Scan 500 (Interscience, Paris, France). Saliva + DTT and the remainder of each DA+DTT were centrifuged at maximum speed (> 13,000 RPM) for 5 min. The supernatant was removed, and 1 mL of sterile Allprotect reagent (Qiagen, Hilden, Germany) was added to the microbial pellet and then stored at − 80 °C.

### DNA isolation

On the day of the DNA isolation, DA pellets were thawed on ice and processed as described previously [28]. Microbial DNA was isolated using the MagAttract PowerSoil DNA KF Kit (Qiagen) on a KingFisher Duo (Thermo Fisher Scientific, Waltham, MA, USA), and quantified using Qubit dsDNA high sensitivity and Qubit dsDNA BR Assay kits (Invitrogen by Thermo Fisher Scientific) on a Qubit 4 Fluorometer (Invitrogen, Carlsbad, CA, USA).

### 16S rRNA gene sequencing

Extracted DNA was amplified, barcoded, and sequenced as described previously [8, 9, 29]. Briefly, amplification of the variable 4 (V4) region of the 16S rRNA gene was performed in 20 μL duplicate reactions with: 8 μL of 2.5× 5Prime Hotstart Mastermix (VWR, Radnor, PA, USA), 1 μL of 20× Evagreen (VWR), 2 μL each of 5 μM forward and reverse primers (519F, barcoded 806R, IDT, CoralVille, IA, USA), 3.5 μL of water, and 3.5 μL of extracted DNA template. A CFX96 RT-PCR machine (Bio-Rad Laboratories, Hercules, CA, USA) was used to monitor amplification reactions and all samples were removed in late exponential phase (~ 10,000 FRU) to minimize chimera formation and non-specific amplification [9, 54, 55]. Amplification was performed under the following cycling conditions: 94 °C for 3 min, up to 50 cycles of 94 °C for 45 s, 54 °C for 60 s, and 72 °C for 90 s. Several samples were rerun after diluting the template as they showed non-exponential amplification in the undiluted sample, a sign of PCR inhibition. Amplified duplicates were pooled together and quantified with KAPA library quantification kit (Roche, Basel, Switzerland) and then all samples were pooled at equimolar concentrations with up to 96 samples per library. AMPureXP beads (Beckman Coulter, Brea, CA, USA) were used to clean up and concentrate libraries before final library quantification with a High Sensitivity D1000 Tapestation Chip (Agilent, Santa Clara, CA, USA). Illumina MiSeq sequencing was performed with a 2 × 300 bp reagent kit by Fulgent Genetics (Temple City, CA, USA).

Raw reads were demultiplexed by Fulgent Genetics. Demultiplexed forward and reverse reads were processed with QIIME 2 2020.2 [56]. Loading of sequence data was performed with the demux plugin followed by quality filtering and denoising with the dada2 plugin [57]. Dada2 trimming parameters were set to the base pair where the average quality score dropped below thirty. All samples were rarefied to the lowest read depth present in all samples (45,386 reads) to decrease biases from varying sequencing depth between samples [58]. The q2-feature-classifier [59] was then used to assign taxonomy to amplicon sequence variants (ASV) with the Silva [60] 132 99% OTUs references. Resulting read count tables were used for downstream analyses in IPython notebooks (see “Data availability” section).

### *Klebsiella*-specific qPCR

Primers specific for the *Klebsiella gltA* gene [61] (F: 5′-CAGGCCGAATATGACGAATTC-3′, R: 5′-CGGGTGATCTGCTCATGAA-3′) were first informatically evaluated for coverage across *Klebsiella pneumoniae*, *Klebsiella oxytoca,* and *Klebsiella aerogenes* via Primer-BLAST [62]. This primer set was found to have a perfect match against strains from all three tested *Klebsiella* species. These primers were also evaluated in the lab for specificity against *Escherichia coli*. No amplification after 40 cycles was observed with a DNA equivalent of ~ 10^6^ *E. coli* cells from the Zymo microbial community DNA standard (Zymo Research, Irvine, CA, USA). *Klebsiella* qPCR was performed in 10 μL reactions with 5 μL of Ssofast Evagreen Supermix (Bio-Rad Laboraties), 0.5 μL of 10 μM *gltA* primers, and 3.5 μL of water. A CFX96 RT-PCR machine (Bio-Rad Laboratories) was used for amplification with the following cycling conditions: 95 °C for 3 min, 40 cycles of 95 °C for 15 s, 62 °C for 30 s, and 68 °C for 30 s. Estimated conversion of cycle threshold (Cq) to copies/μL was performed where a Cq of 22.4 equals 1000 copies/uL. *Klebsiella* load was then calculated by adjusting for dilutions and normalizing to the collected sample volume.

### Absolute abundance

The total microbial load (bacteria and archaea) of each sample and the absolute abundance of each taxon in individual samples was determined as described previously [9, 29]. Briefly, the Bio-Rad QX200 droplet dPCR system (Bio-Rad Laboratories) was utilized to measure the 16S concentration in each sample with the following reaction components: 1X QX200 EvaGreen Supermix (Bio-Rad), 500 nM forward primer, and 500 nM reverse primer (519F, 806R) and thermocycling conditions: 95 °C for 5 min, 40 cycles of 95 °C for 30 s, 52 °C for 30 s, and 68 °C for 30 s, followed by a dye stabilization step of 4 °C for 5 min and 90 °C for 5 min. The final concentration of 16S rRNA gene copies in each sample was corrected for dilutions and normalized to the extracted sample volume.

For each sample, the input-volume-normalized total microbial load from dPCR was multiplied by each amplicon sequence variant’s (ASV) relative abundance to determine the absolute abundance of each ASV. No correlation between collected sample volume and measured bacterial load was observed. The average of all sample volumes for a specific sample type was used for a few samples (11 duodenum, 10 saliva) that were missing the starting volume information. A 95% confidence interval of input volumes for duodenum samples ranged from 0.18 to 1.93 mL indicating that the estimated input volume measurement would likely be up to 4× off in either direction while the total microbial load ranged 40,000X. Similarly, a 95% confidence interval of input volumes for saliva samples ranged from 0.36 to 1.28 mL indicating that the estimated input volume measurement would likely be up to 2× off in either direction while the total microbial load ranged 82X.

### Poisson quality filtering

Two separate quality-filtering steps based on Poisson statistics were used to determine the statistical confidence in the measured values. First, a 95% confidence interval was calculated from the repeated measures of water blanks. Samples with a total microbial load below the upper bound of this confidence interval were removed from further analysis.

Second, the limit of detection (LOD) in terms of relative abundance was determined for each sample. Sequencing can be divided into two separate Poisson sampling steps. First, an aliquot of sample is taken from the extracted sample and input into the library amplification reaction. The LOD of the library amplification step was determined by multiplying the total microbial load from dPCR by the input volume into the library amplification reaction and then finding the relative abundance corresponding to an input of three copies. Poisson statistics tells us that the likelihood of sampling one or more copies with an average input of three copies is 95%. The second Poisson sampling step in sequencing arises from the number of reads generated from the amplified library. The accuracy of the second Poisson sampling step was previously shown [9] to follow a negative exponential curve, LOD = 7.115 ∗ read depth^−0.115^, between the total read depth and relative abundance at which 95% confidence of detection is observed. The minimum of the two described LODs (first determined per sample by total load, and second by sequencing depth) was then determined for each sample. For each sample, the abundance of any ASV with a relative abundance below the LOD was set to zero. After filtering, data tables for each taxonomic level were generated.

### Data transforms and dimensionality reduction

For PCA, all absolute taxon abundances were log-transformed. To handle zeros, a pseudo-count of 0.1 reads was added to all taxon relative abundances before multiplying by each sample’s total microbial load as determined by digital PCR. PCA was performed with the *sklearn.decomposition.PCA* function in Python. Ranked feature loadings for each taxon on a given principal component were determined by scaling the corresponding eigenvector by the maximum transformed value for that principal component axis.

### Statistical analysis and correlations

Group comparisons (e.g., SIBO vs. no SIBO, saliva vs. duodenum) were analyzed using the non-parametric Kruskal-Wallis rank sums tests with Benjamini–Hochberg multiple hypothesis testing correction using *SciPy.stats Kruskal* function and *statsmodels.stats.multitest multipletests* function with the *fdr_bh* option.

Correlation coefficients were either Spearman or Pearson and corresponding *P* values for all correlations were determined with *scipy.stats.spearmanr* or *scipy.stats.pearsonr* functions. Multiple hypothesis testing was performed for each group of correlations (e.g., taxa co-correlations, cytokine correlations) separately using the Benjamini–Hochberg procedure.

## Supplementary Information

**Additional file 1: Figure S1.** Total microbial load breakdown by age (A) and gender (B). **Figure S2.** Distribution of total microbial load from subpopulations of patients: taking probiotics (N=49), active smokers (N=16), taking antibiotics in the past 6 months (N=100), or taking proton pump inhibitors (PPI, N=106). **Figure S3.** (A) Scatterplot comparing aerobic culture load from MacConkey plates to total load from 16S quantitative sequencing of only the subset of bacteria that are known to grow on MacConkey plates (*Escherichia-Shigella*, *Enterobacteriaceae*, *Enterococcus*, and *Aeromonas*)^1^. (B) Scatterplot comparing anaerobic culture load, from blood agar plates, to total load from sequencing of prevalent bacteria that are expected to grow on blood agar plates (*Prevotella*, *Streptococcus*, *Fusobacterium*, *Escherichia-Shigella*)^2^. Red dashed line indicates limit of detection of quantitative sequencing method. N = 244. (Six patients in the study were lacking culture data). **Figure S4.** (A) Cycle threshold (Cq) values yielded by qPCR with *Klebsiella*-specific primers. Duodenum aspirate samples were classified via quantitative sequencing as containing *Enterobacteriaceae* (“*Entero* +”, N=22) or not containing *Enterobacteriaceae* (“*Entero* –”, N=8). (B) Total loads of *Enterobacteriaceae* (copies/mL) in duodenum aspirates as a factor of the approximate *Klebsiella load* (copies/mL). *Enterobacteriaceae* measurements are calculated based on 16S rRNA gene copies (8 copies/genome) and *Klebsiella* measurements are calculated based on the citrate synthase gene (gltA, 1 copy/genome). **Figure S5**. Receiver operating characteristic (ROC) curve using absolute loads of seven disruptor taxa (*Enterobacteriaceae*, *Escherichia-Shigella*, *Clostridium sensu stricto 1*, *Enterococcus*, *Romboutsia*, *Aeromonas*, *Bacteroides*) identified in the sequencing data for SIBO classification. SIBO classification was made based on gold-standard aerobic culture results, ≥10^3^ CFU/mL of duodenal aspirate. Data points are connected by a line between each consecutive point. **Figure S6.** IL8 levels in samples with and without a *Clostridium* which, based on the V4 region of the 16S rRNA gene, was classified as *C. perfringens*. **Figure S7.** Relationship between *Lactobacillus* load and bloating symptoms in samples containing additional (non-*Lactobacillus*) disruptor taxa. **Figure S8.** Violin plots with data points overlaid for patient-reported symptom scores. Binary threshold for determining whether severe symptoms exist was set at the median score reported of each symptom, shown by the red-dashed lines. **Figure S9.** Disruptor taxa load separated by patient age: 18-39 (N=40), 40-49 (N=31), 50-59 (N=58), 60-69 (N=67), 70-83 (N=54). **Figure S10.** Relationship between absolute abundance (greater than 10^5^ copies/mL) and relative abundance of disruptor loads (Spearman, *P*=0.09, not significant). **Figure S11**. Comparison of total microbial load between human duodenum, mouse duodenum, and mouse duodenum where the mice had coprophagy prevented via tail cup. Mouse data from Bogatyrev et al. 2020^3^. Reported *P*-values are from Kruskal-Wallis test. **Table S1.** Summary statistics for the patient cohort used in this study. All patients are from the REIMAGINE study^4^. **Table S2.** *P*-values from significance tests (Kruskal-Wallis) comparing total microbial load between selected subgroups of individuals. Significance is indicated with an asterisk. **Table S3.** Comparison between prevalence and relative abundance of all taxa in paired saliva and duodenum samples (N=21 participants). **Table S4.** Two groups of taxa (light blue and dark blue) that have stronger co-correlations with another taxon than with total load. Significance values for all correlations and co-correlations were *P* < 0.001.

**Additional file 2.**

## Data Availability

Sequencing data generated during this study are available in the National Center for Biotechnology Information Sequence Read Archive repository under study accession number PRJNA674353. Raw data for each figure and IPython notebooks for data processing and figure generation are available through CaltechDATA: https://data.caltech.edu/records/1701.
