# Supplementary material for: Quantitative sequencing clarifies the role of disruptor taxa, oral microbiota, and strict anaerobes in the human small-intestine microbiome
Source: Microbiome. 2021 Nov 2;9:214. doi: 10.1186/s40168-021-01162-2 (PMC8561862; doi:10.1186/s40168-021-01162-2)
Supplement: Supplementary file 2 — Additional file 1: Figure S1. Total microbial load breakdown by age (A) and gender (B). Figure S2. Distribution of total microbial load from subpopulations of patients: taking probiotics (N=49), active smokers (N=16), taking antibiotics in the past 6 months (N=100), or taking proton pump inhibitors (PPI, N=106). Figure S3. (A) Scatterplot comparing aerobic culture load from MacConkey plates to total load from 16S quantitative sequencing of only the subset of bacteria that are known to grow on MacConkey plates (Escherichia-Shigella, Enterobacteriaceae, Enterococcus, and Aeromonas)1. (B) Scatterplot comparing anaerobic culture load, from blood agar plates, to total load from sequencing of prevalent bacteria that are expected to grow on blood agar plates (Prevotella, Streptococcus, Fusobacterium, Escherichia-Shigella)2. Red dashed line indicates limit of detection of quantitative sequencing method. N = 244. (Six patients in the study were lacking culture data). Figure S4. (A) Cycle threshold (Cq) values yielded by qPCR with Klebsiella-specific primers. Duodenum aspirate samples were classified via quantitative sequencing as containing Enterobacteriaceae (“Entero +”, N=22) or not containing Enterobacteriaceae (“Entero –”, N=8). (B) Total loads of Enterobacteriaceae (copies/mL) in duodenum aspirates as a factor of the approximate Klebsiella load (copies/mL). Enterobacteriaceae measurements are calculated based on 16S rRNA gene copies (8 copies/genome) and Klebsiella measurements are calculated based on the citrate synthase gene (gltA, 1 copy/genome). Figure S5. Receiver operating characteristic (ROC) curve using absolute loads of seven disruptor taxa (Enterobacteriaceae, Escherichia-Shigella, Clostridium sensu stricto 1, Enterococcus, Romboutsia, Aeromonas, Bacteroides) identified in the sequencing data for SIBO classification. SIBO classification was made based on gold-standard aerobic culture results, ≥103 CFU/mL of duodenal aspirate. Data points are connected by a lin [file 40168_2021_1162_MOESM2_ESM.docx]

Supplementary Info

**Quantitative sequencing clarifies the role of disruptor taxa, the oral microbiota, and strict anaerobes in the human small-intestine microbiome**

Jacob T. Barlow^1, 6^, Gabriela Leite^2, 6^, Anna E. Romano^3^, Rashin Sedighi^2^, Christine Chang^2^, Shreya Celly^2^, Ali Rezaie^2, 4^, Ruchi Mathur^2, 5^, Mark Pimentel^2, 4^, Rustem F. Ismagilov^1,3^*

^1^ Division of Biology and Biological Engineering, California Institute of Technology, Pasadena, CA 91125, USA

^2^ Medically Associated Science and Technology (MAST) Program, Cedars-Sinai Medical Center, Los Angeles, CA 90048, USA

^3^ Division of Chemistry and Chemical Engineering, California Institute of Technology, Pasadena, CA 91125, USA

^4^ Division of Digestive and Liver Diseases, Cedars-Sinai Medical Center, Los Angeles, CA 90048, USA

^5^ Division of Endocrinology, Diabetes, and Metabolism, Cedars-Sinai Medical Center, Los Angeles, CA 90048, USA

*Correspondence: rustem.admin@caltech.edu

^6^ These authors contributed equally

Figures S1-S11

Tables S1-S4

Detailed Contributions of Non-corresponding Authors

Supplementary References


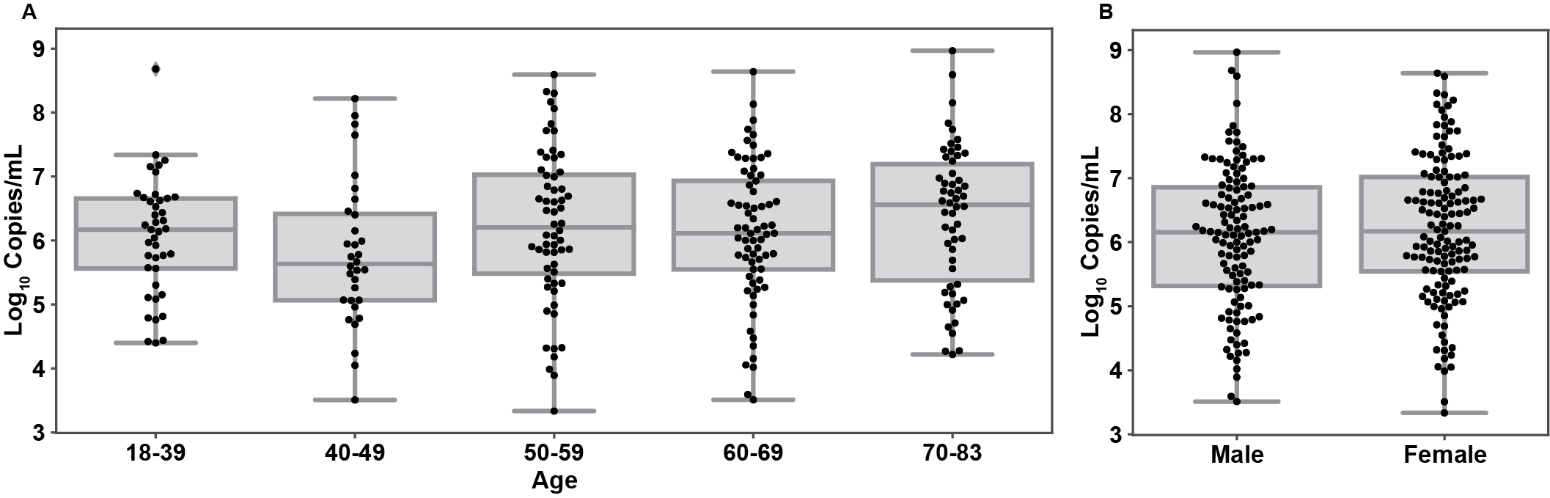
**Figure S1:** Total microbial load breakdown by age (A) and gender (B).


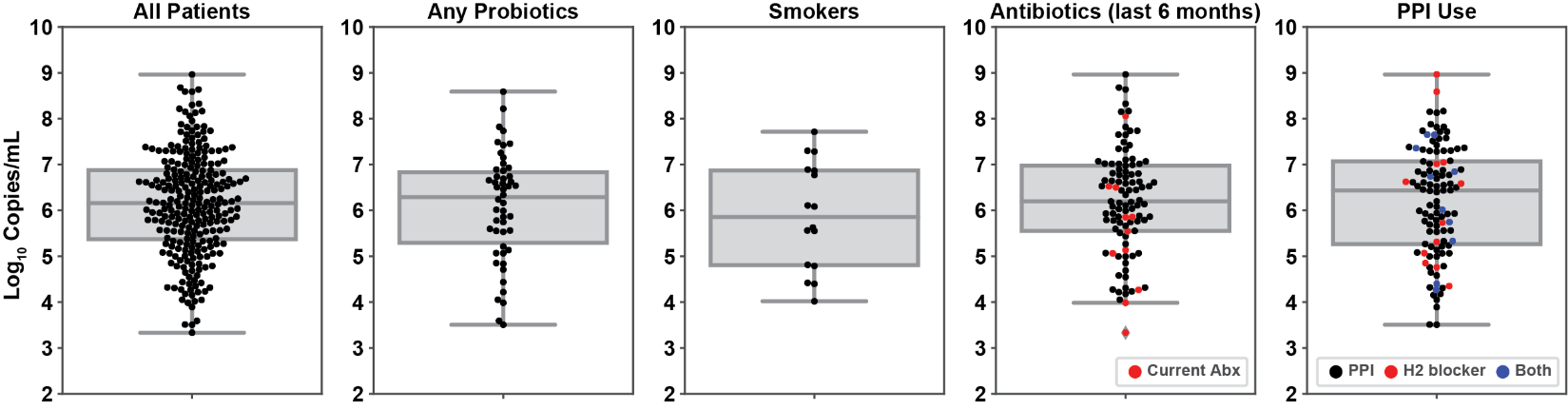


**Figure S2:** Distribution of total microbial load from subpopulations of patients: taking probiotics (N=49), active smokers (N=16), taking antibiotics in the past 6 months (N=100), or taking proton pump inhibitors (PPI, N=106).

**
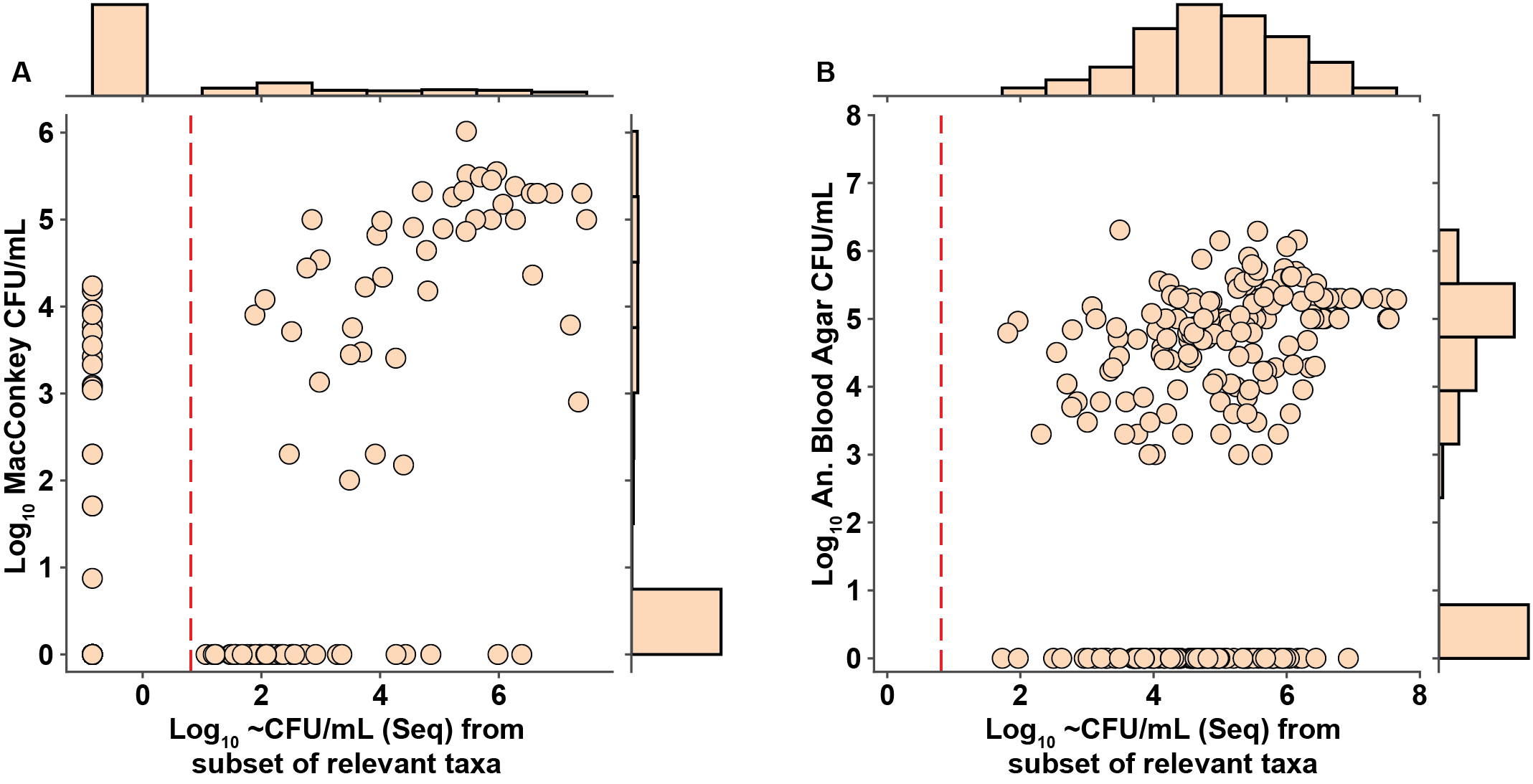
Figure S3**: (A) Scatterplot comparing aerobic culture load from MacConkey plates to total load from 16S quantitative sequencing of only the subset of bacteria that are known to grow on MacConkey plates (*Escherichia-Shigella*, *Enterobacteriaceae*, *Enterococcus*, and *Aeromonas*)^1^. (B) Scatterplot comparing anaerobic culture load, from blood agar plates, to total load from sequencing of prevalent bacteria that are expected to grow on blood agar plates (*Prevotella*, *Streptococcus*, *Fusobacterium*, *Escherichia-Shigella*)^2^. Red dashed line indicates limit of detection of quantitative sequencing method. N = 244. (Six patients in the study were lacking culture data.)


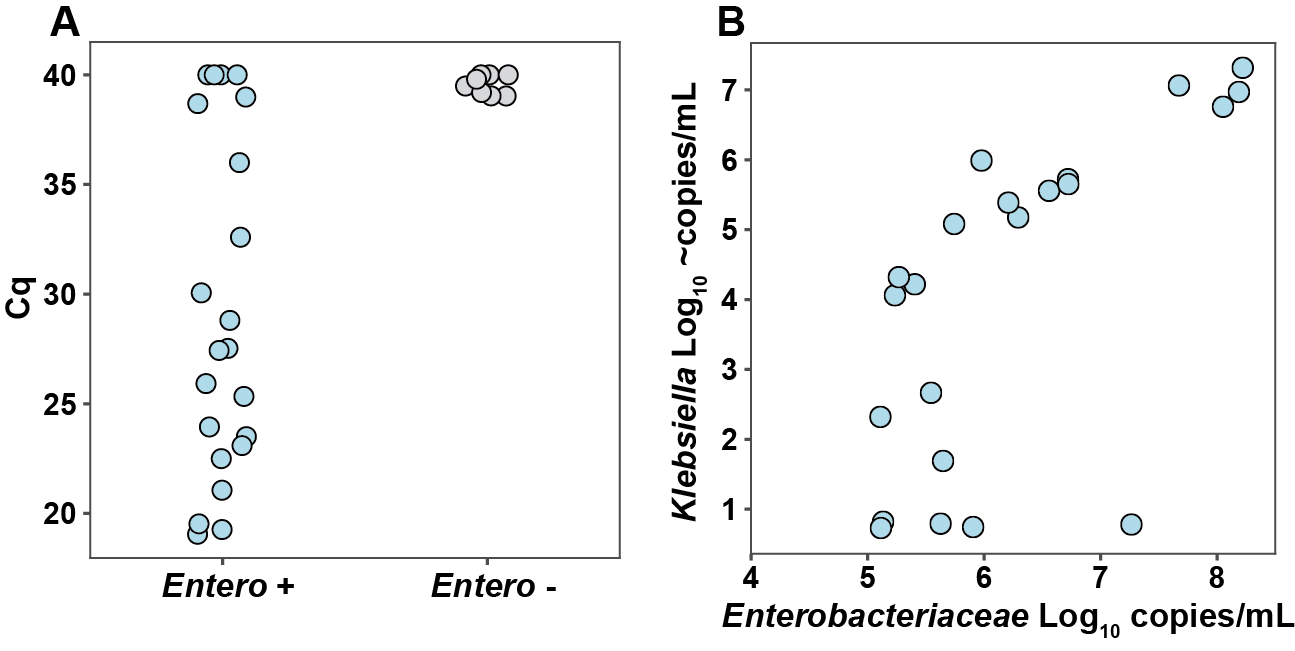


**Figure S4:** (A) Cycle threshold (Cq) values yielded by qPCR with *Klebsiella*-specific primers. Duodenum aspirate samples were classified via quantitative sequencing as containing *Enterobacteriaceae* (“*Entero* +”, N=22) or not containing *Enterobacteriaceae* (“*Entero* –”, N=8). (B) Total loads of *Enterobacteriaceae* (copies/mL) in duodenum aspirates as a factor of the approximate *Klebsiella load* (copies/mL). *Enterobacteriaceae* measurements are calculated based on 16S rRNA gene copies (8 copies/genome) and *Klebsiella* measurements are calculated based on the citrate synthase gene (gltA, 1 copy/genome).


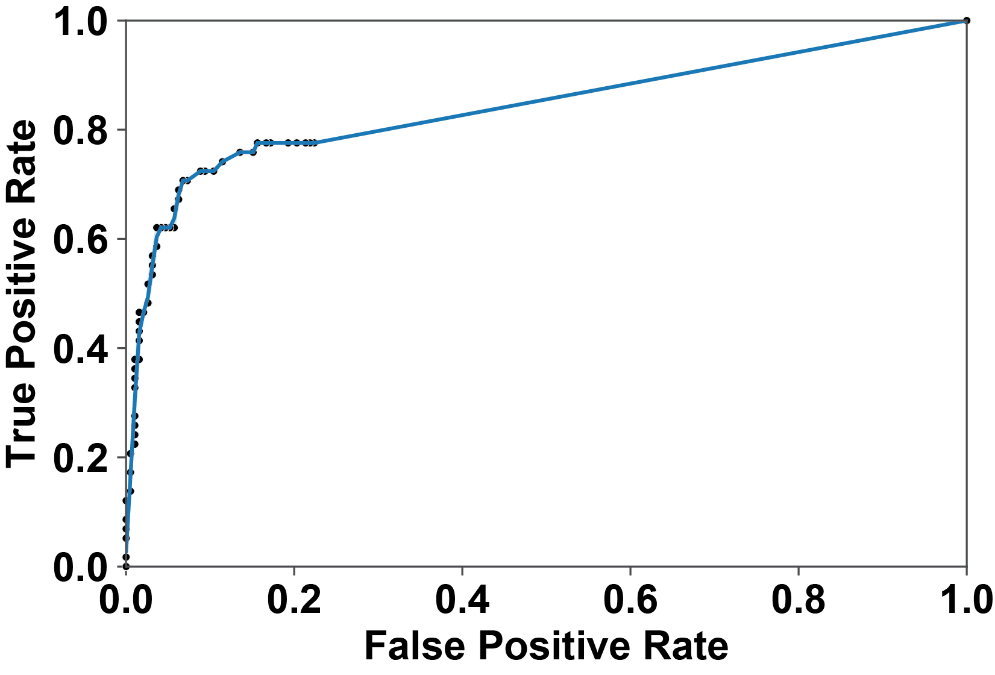


**Figure S5**: Receiver operating characteristic (ROC) curve using absolute loads of seven disruptor taxa (*Enterobacteriaceae*, *Escherichia-Shigella*, *Clostridium sensu stricto 1*, *Enterococcus*, *Romboutsia*, *Aeromonas*, *Bacteroides*) identified in the sequencing data for SIBO classification. SIBO classification was made based on gold-standard aerobic culture results, ≥10^3^ CFU/mL of duodenal aspirate. Data points are connected by a line between each consecutive point.


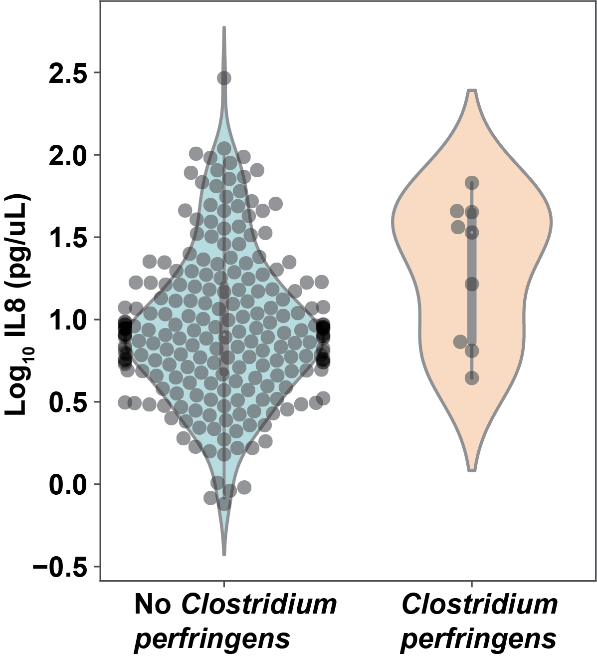


**Figure S6:** IL8 levels in samples with and without a *Clostridium* which, based on the V4 region of the 16S rRNA gene, was classified as *C. perfringens*.


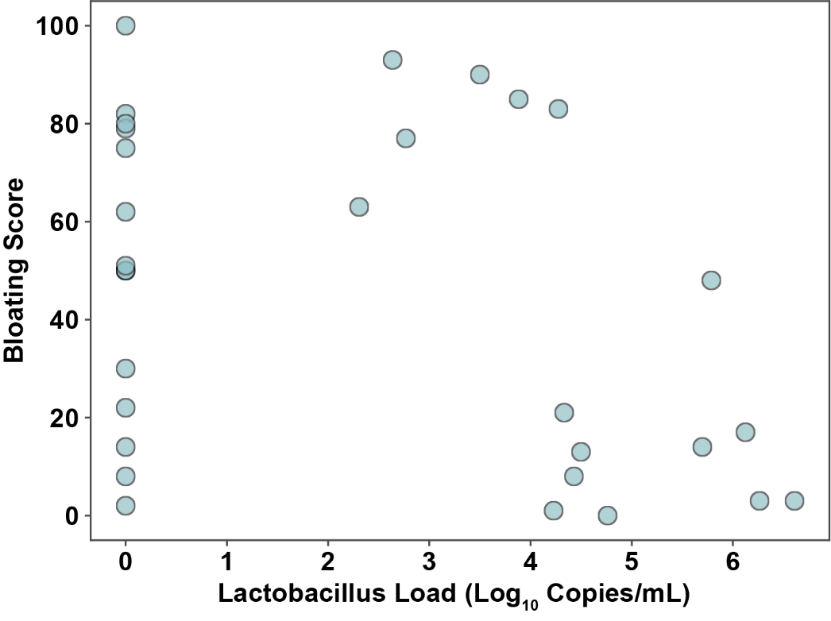


**Figure S7:** Relationship between *Lactobacillus* load and bloating symptoms in samples containing additional (non-*Lactobacillus*) disruptor taxa.


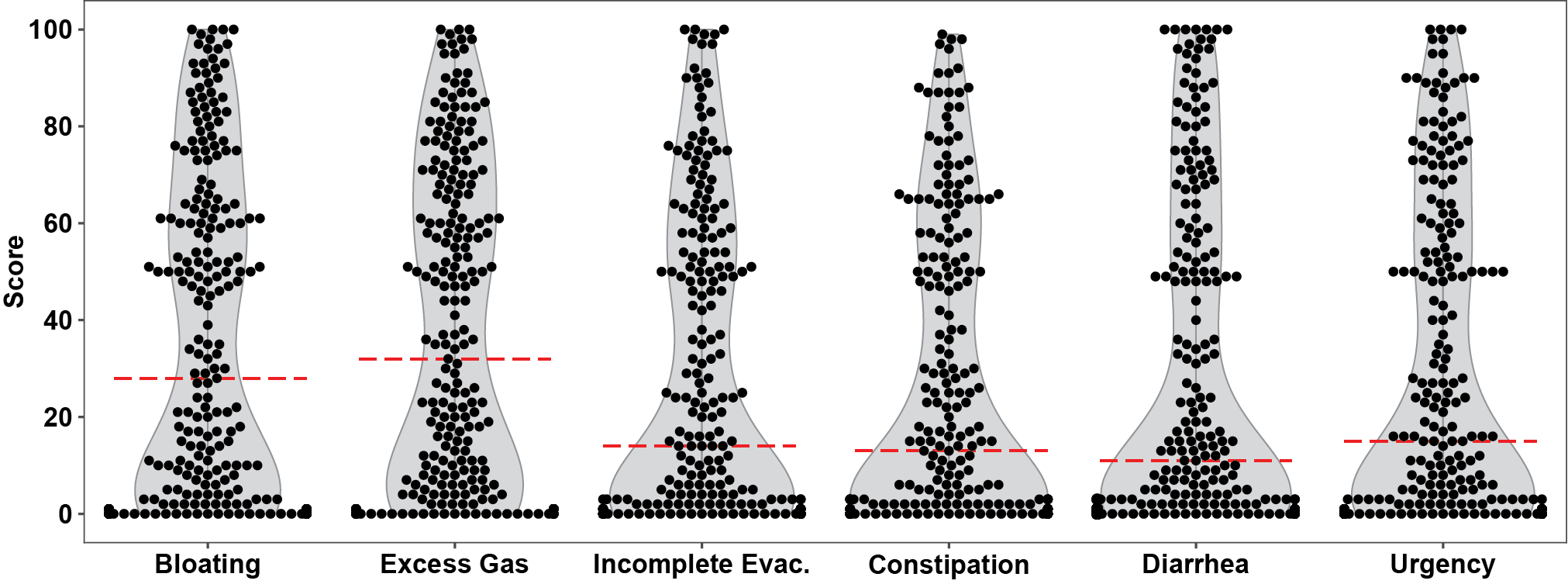


**Figure S8:** Violin plots with data points overlaid for patient-reported symptom scores. Binary threshold for determining whether severe symptoms exist was set at the median score reported of each symptom, shown by the red-dashed lines.

**
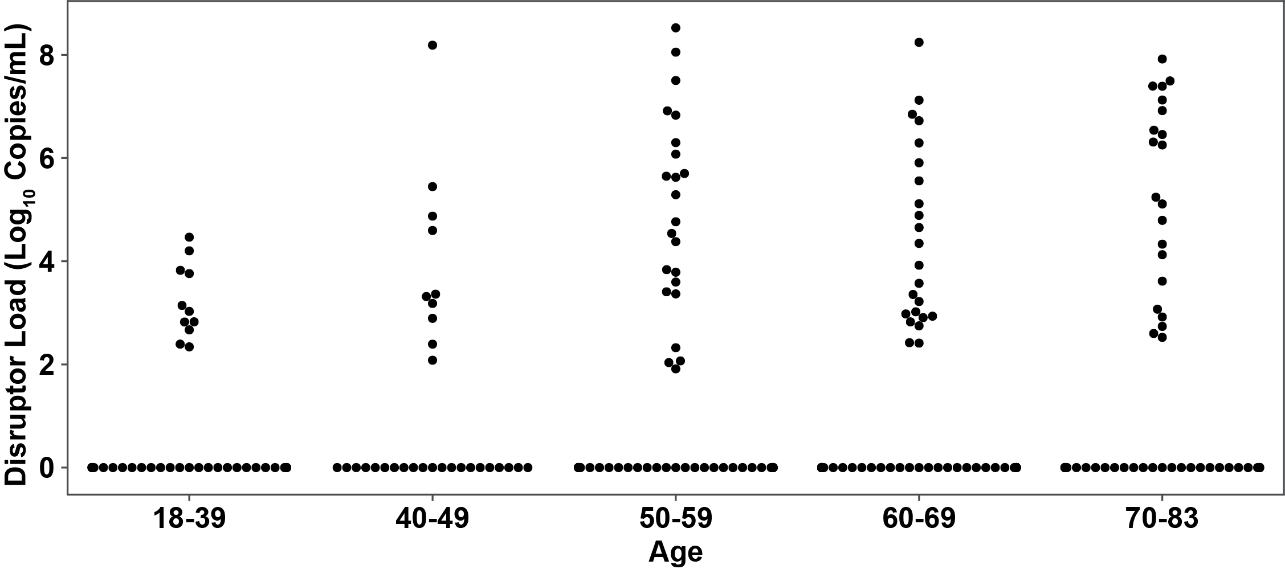
Figure S9**: Disruptor taxa load separated by patient age: 18-39 (N=40), 40-49 (N=31), 50-59 (N=58), 60-69 (N=67), 70-83 (N=54).


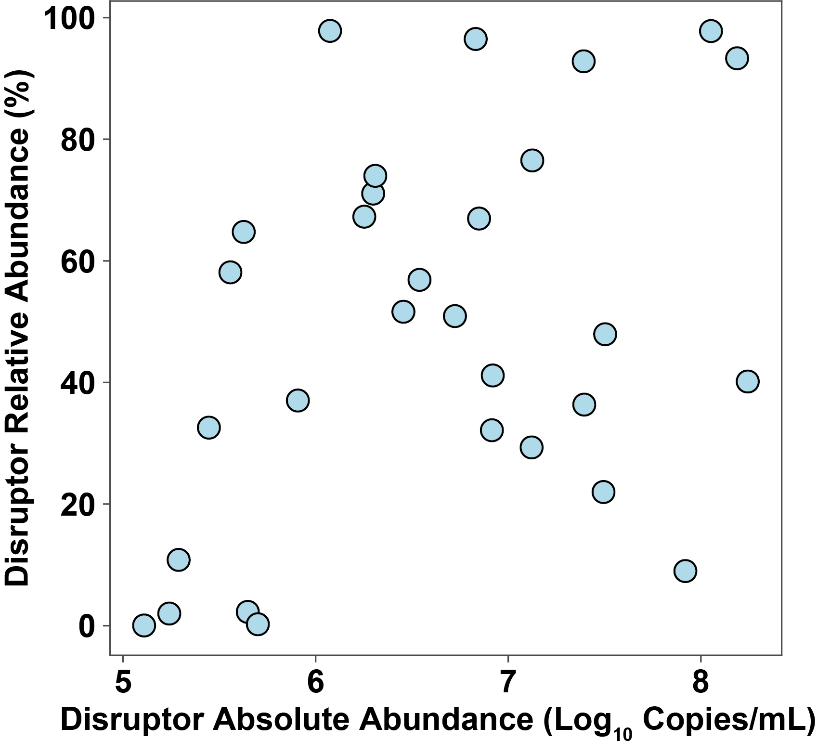


**Figure S10:** Relationship between absolute abundance (greater than 10^5^ copies/mL) and relative abundance of disruptor loads (Spearman, *P*=0.09, not significant)

**
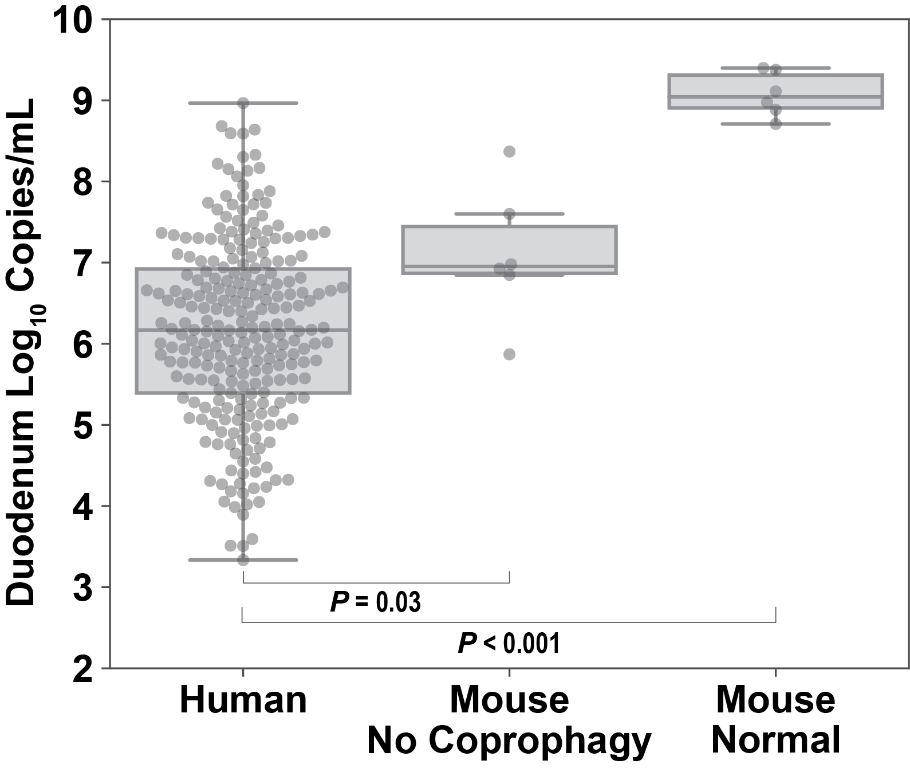
**

**Figure S11:** Comparison of total microbial load between human duodenum, mouse duodenum, and mouse duodenum where the mice had coprophagy prevented via tail cup. Mouse data from Bogatyrev et al. 2020^3^. Reported *P*-values are from Kruskal-Wallis test.

**Table S1:** Summary statistics for the patient cohort used in this study. All patients are from the REIMAGINE study^4^.

| **Total subjects** |  | **Total** | **SIBO** | **non-SIBO** |
| --- | --- | --- | --- | --- |
|  | **Duodenal Aspirate** | 250 | 23% (58) | 77% (192) |
|  | **Saliva** | 21 | 19% (4) | 81% (17) |
|  |  | **Mean (Std Dev)** |  |  |
|  |  | **Total** | **SIBO** | **non-SIBO** |
| **Age** |  | 56.9 (14.9) | 61.6 (13.6) | 55.5 (15.0) |
| **Weight (lbs)** |  | 169.4 (49.5) | 166.1 (38.7) | 170.4 (52.4) |
|  |  | **Percent (N)** |  |  |
| **Gender** |  | **Total** | **SIBO** | **non-SIBO** |
|  | **Male** | 46% (115) | 38% (22) | 48% (93) |
|  | **Female** | 54% (135) | 62% (36) | 52% (99) |
| **Antibiotic usage** |  |  |  |  |
|  | **last 6 months** | 40% (100) | 59% (34) | 34% (66) |
|  | **current** | 4% (11) | 9% (5) | 3% (6) |
| **PPI usage** |  |  |  |  |
|  | **PPI** | 34% (86) | 36% (21) | 34% (65) |
|  | **H2 blocker** | 4% (10) | 7% (4) | 3% (6) |
|  | **both** | 4% (10) | 5% (3) | 4% (7) |
| **Any probiotic usage** |  | 20% (49) | 26% (15) | 18% (34) |
| **Smokers** |  | 6% (16) | 3% (2) | 7% (14) |
| **Symptom Scores** |  |  |  |  |
|  | **Bloating > 50^th^ percentile** | 50% | 57% (33) | 45% (86) |
|  | **Constipation > 50^th^ percentile** | 50% | 53% (31) | 46% (89) |
|  | **Incomplete Evacuation > 50^th^ percentile** | 50% | 55% (32) | 47% (91) |
|  | **Excess Gas > 50^th^ percentile** | 50% | 57% (33) | 45% (86) |
|  | **Diarrhea > 50^th^ percentile** | 50% | 55% (32) | 46% (88) |
| **Reason for Endoscopy** |  |  |  |  |
|  | **GERD/dyspepsia workup** | 21% (53) | 16% (9) | 23% (44) |
|  | **Possible bleeding/anemia workup** | 7% (17) | 10% (6) | 6% (11) |
|  | **Rule out cancer/polyp** | 24% (59) | 29% (17) | 22% (42) |
|  | **Biliary disease** | 13% (32) | 16% (9) | 12% (23) |
|  | **Dysphagia** | 10% (24) | 9% (5) | 10% (19) |
|  | **Crohn’s disease** | 4% (10) | 3% (2) | 4% (8) |
|  | **Functional GI disease** | 5% (13) | 2% (1) | 6% (12) |
|  | **Rule out Celiac disease** | 1% (3) | 3% (2) | 1% (1) |
|  | **Known peptic ulcer disease** | 1% (3) | 0% (0) | 2% (3) |
|  | **G-tube management** | 1% (2) | 2% (1) | 1% (1) |
|  | **Other** | 3% (8) | 2% (1) | 4% (7) |
|  | **Missing Information** | 10% (26) | 9% (5) | 11% (21) |

**Table S2:** *P*-values from significance tests (Kruskal-Wallis) comparing total microbial load between selected subgroups of individuals. Significance is indicated with an asterisk.

| **Comparison (N)** | **p-value** |
| --- | --- |
| Taking probiotics (49) vs no probiotics (201) | 0.97 |
| Abx past 6 months (100) vs no Abx past 6 months (150) | 0.67 |
| Current Abx (11) vs not currently taking Abx (239) | **0.04*** |
| Current Abx (11) vs Abx past 6 months (100) | **0.02*** |
| Taking PPI (106) vs no PPI (144) | 0.29 |
| Current smoker (16) vs not currently smoker (234) | 0.39 |

**Table S3:** Comparison between prevalence and relative abundance of all taxa in paired saliva and duodenum samples (N=21 participants).

| **# of Saliva samples taxon appears in (N=21 total)** | **# of Duodenum samples taxon appears in (N=21 total)** | **Saliva Rel. Abundance (%)** | **Duodenum Rel. Abundance (%)** | **Taxonomy** | **Difference between # of samples taxon is present in between Saliva and Duodenum (N=21 total)** |
| --- | --- | --- | --- | --- | --- |
| 21 | 8 | 0.22 | 0.20 | D_0__Bacteria;D_1__Bacteroidetes;D_2__Bacteroidia;D_3__Flavobacteriales;D_4__Weeksellaceae;D_5__Bergeyella;D_6__uncultured bacterium | 13 |
| 19 | 6 | 0.21 | 0.09 | D_0__Bacteria;D_1__Bacteroidetes;D_2__Bacteroidia;D_3__Bacteroidales;D_4__Prevotellaceae;D_5__Prevotella;__ | 13 |
| 16 | 6 | 0.27 | 0.05 | D_0__Bacteria;D_1__Firmicutes;D_2__Negativicutes;D_3__Selenomonadales;D_4__Veillonellaceae;D_5__Selenomonas 3;__ | 10 |
| 18 | 8 | 1.63 | 0.49 | D_0__Bacteria;D_1__Bacteroidetes;D_2__Bacteroidia;D_3__Bacteroidales;D_4__Prevotellaceae;D_5__Alloprevotella;D_6__uncultured Bacteroidetes bacterium | 10 |
| 20 | 10 | 0.93 | 0.07 | D_0__Bacteria;D_1__Epsilonbacteraeota;D_2__Campylobacteria;D_3__Campylobacterales;D_4__Campylobacteraceae;D_5__Campylobacter;__ | 10 |
| 15 | 5 | 0.32 | 0.05 | D_0__Bacteria;D_1__Bacteroidetes;D_2__Bacteroidia;D_3__Flavobacteriales;D_4__Flavobacteriaceae;D_5__Capnocytophaga;__ | 10 |
| 12 | 3 | 0.04 | 0.01 | D_0__Bacteria;D_1__Bacteroidetes;D_2__Bacteroidia;D_3__Flavobacteriales;D_4__Flavobacteriaceae;D_5__Capnocytophaga;D_6__Capnocytophaga gingivalis | 9 |
| 12 | 3 | 0.13 | 0.01 | D_0__Bacteria;D_1__Proteobacteria;D_2__Gammaproteobacteria;D_3__Betaproteobacteriales;D_4__Burkholderiaceae;D_5__Lautropia;D_6__uncultured bacterium | 9 |
| 15 | 6 | 0.28 | 0.05 | D_0__Bacteria;D_1__Bacteroidetes;D_2__Bacteroidia;D_3__Bacteroidales;D_4__Prevotellaceae;D_5__Prevotella;D_6__Prevotella oris | 9 |
| 13 | 4 | 0.09 | 0.04 | D_0__Bacteria;D_1__Proteobacteria;D_2__Gammaproteobacteria;D_3__Cardiobacteriales;D_4__Cardiobacteriaceae;D_5__Cardiobacterium;D_6__uncultured bacterium | 9 |
| 16 | 8 | 0.96 | 0.16 | D_0__Bacteria;D_1__Proteobacteria;D_2__Gammaproteobacteria;D_3__Betaproteobacteriales;D_4__Neisseriaceae;D_5__Neisseria;__ | 8 |
| 11 | 3 | 0.56 | 0.07 | D_0__Bacteria;D_1__Bacteroidetes;D_2__Bacteroidia;D_3__Bacteroidales;D_4__Prevotellaceae;D_5__Alloprevotella;__ | 8 |
| 13 | 6 | 0.54 | 0.21 | D_0__Bacteria;D_1__Firmicutes;D_2__Bacilli;D_3__Lactobacillales;D_4__Streptococcaceae;D_5__Streptococcus;D_6__Streptococcus mutans | 7 |
| 16 | 9 | 1.73 | 0.37 | D_0__Bacteria;D_1__Bacteroidetes;D_2__Bacteroidia;D_3__Bacteroidales;D_4__Prevotellaceae;D_5__Prevotella;D_6__Prevotella sp. oral taxon 299 str. F0039 | 7 |
| 14 | 7 | 0.10 | 0.02 | D_0__Bacteria;D_1__Firmicutes;D_2__Clostridia;D_3__Clostridiales;D_4__Family XIII;D_5__[Eubacterium] nodatum group;D_6__[Eubacterium] sulci | 7 |
| 12 | 5 | 0.09 | 0.01 | D_0__Bacteria;D_1__Actinobacteria;D_2__Actinobacteria;D_3__Corynebacteriales;D_4__Corynebacteriaceae;D_5__Corynebacterium;__ | 7 |
| 12 | 5 | 0.08 | 0.03 | D_0__Bacteria;D_1__Bacteroidetes;D_2__Bacteroidia;D_3__Bacteroidales;D_4__Prevotellaceae;D_5__Prevotella 7;D_6__Prevotella sp. oral clone P4PB_83 P2 | 7 |
| 20 | 13 | 2.59 | 1.61 | D_0__Bacteria;D_1__Bacteroidetes;D_2__Bacteroidia;D_3__Bacteroidales;D_4__Porphyromonadaceae;D_5__Porphyromonas;__ | 7 |
| 10 | 3 | 0.09 | 0.02 | D_0__Bacteria;D_1__Epsilonbacteraeota;D_2__Campylobacteria;D_3__Campylobacterales;D_4__Campylobacteraceae;D_5__Campylobacter;D_6__Campylobacter rectus | 7 |
| 8 | 1 | 0.06 | 0.00 | D_0__Bacteria;D_1__Proteobacteria;D_2__Gammaproteobacteria;D_3__Betaproteobacteriales;D_4__Neisseriaceae;D_5__Kingella;D_6__uncultured bacterium | 7 |
| 14 | 8 | 0.13 | 0.16 | D_0__Bacteria;D_1__Firmicutes;D_2__Bacilli;D_3__Lactobacillales;D_4__Streptococcaceae;D_5__Streptococcus;D_6__Streptococcus anginosus subsp. anginosus | 6 |
| 17 | 11 | 0.18 | 0.30 | D_0__Bacteria;D_1__Firmicutes;D_2__Clostridia;D_3__Clostridiales;D_4__Lachnospiraceae;D_5__Oribacterium;__ | 6 |
| 21 | 15 | 1.12 | 0.74 | D_0__Bacteria;D_1__Actinobacteria;D_2__Actinobacteria;D_3__Micrococcales;D_4__Micrococcaceae;D_5__Rothia;D_6__uncultured bacterium | 6 |
| 7 | 1 | 0.06 | 0.03 | D_0__Bacteria;D_1__Bacteroidetes;D_2__Bacteroidia;D_3__Bacteroidales;D_4__Prevotellaceae;D_5__Alloprevotella;D_6__Alloprevotella tannerae | 6 |
| 7 | 1 | 0.02 | 0.00 | D_0__Bacteria;D_1__Bacteroidetes;D_2__Bacteroidia;D_3__Bacteroidales;D_4__Prevotellaceae;D_5__Prevotella;D_6__Prevotella sp. oral taxon G60 | 6 |
| 17 | 11 | 0.30 | 0.20 | D_0__Bacteria;D_1__Actinobacteria;D_2__Actinobacteria;D_3__Corynebacteriales;D_4__Corynebacteriaceae;D_5__Corynebacterium;D_6__Corynebacterium durum | 6 |
| 0 | 6 | 0.00 | 0.26 | D_0__Bacteria;D_1__Firmicutes;D_2__Bacilli;D_3__Lactobacillales;D_4__Streptococcaceae;D_5__Streptococcus;D_6__Streptococcus pneumoniae | 6 |
| 19 | 13 | 0.45 | 0.24 | D_0__Bacteria;D_1__Actinobacteria;D_2__Coriobacteriia;D_3__Coriobacteriales;D_4__Atopobiaceae;D_5__Atopobium;D_6__uncultured bacterium | 6 |
| 9 | 4 | 0.06 | 0.02 | D_0__Bacteria;D_1__Bacteroidetes;D_2__Bacteroidia;D_3__Bacteroidales;D_4__Paludibacteraceae;D_5__F0058;D_6__uncultured bacterium | 5 |
| 7 | 2 | 0.06 | 0.01 | D_0__Bacteria;D_1__Bacteroidetes;D_2__Bacteroidia;D_3__Bacteroidales;D_4__Prevotellaceae;D_5__Prevotella 7;D_6__Prevotella sp. oral clone DO014 | 5 |
| 11 | 6 | 0.15 | 0.05 | D_0__Bacteria;D_1__Proteobacteria;D_2__Gammaproteobacteria;D_3__Betaproteobacteriales;D_4__Neisseriaceae;D_5__Eikenella;D_6__uncultured bacterium | 5 |
| 17 | 12 | 1.79 | 0.45 | D_0__Bacteria;D_1__Bacteroidetes;D_2__Bacteroidia;D_3__Bacteroidales;D_4__Prevotellaceae;D_5__Prevotella 6;D_6__uncultured bacterium | 5 |
| 8 | 3 | 0.04 | 0.02 | D_0__Bacteria;D_1__Bacteroidetes;D_2__Bacteroidia;D_3__Bacteroidales;D_4__Tannerellaceae;D_5__Tannerella;D_6__uncultured bacterium | 5 |
| 19 | 14 | 0.82 | 0.60 | D_0__Bacteria;D_1__Fusobacteria;D_2__Fusobacteriia;D_3__Fusobacteriales;D_4__Leptotrichiaceae;D_5__Leptotrichia;__ | 5 |
| 13 | 8 | 0.30 | 0.14 | D_0__Bacteria;D_1__Firmicutes;D_2__Clostridia;D_3__Clostridiales;D_4__Peptostreptococcaceae;D_5__Peptostreptococcus;D_6__uncultured organism | 5 |
| 11 | 6 | 0.09 | 0.11 | D_0__Bacteria;D_1__Firmicutes;D_2__Bacilli;D_3__Lactobacillales;D_4__Aerococcaceae;D_5__Abiotrophia;D_6__uncultured bacterium | 5 |
| 19 | 14 | 0.51 | 0.33 | D_0__Bacteria;D_1__Firmicutes;D_2__Clostridia;D_3__Clostridiales;D_4__Lachnospiraceae;D_5__Stomatobaculum;D_6__uncultured bacterium | 5 |
| 18 | 13 | 0.33 | 0.39 | D_0__Bacteria;D_1__Firmicutes;D_2__Clostridia;D_3__Clostridiales;D_4__Lachnospiraceae;D_5__Lachnoanaerobaculum;D_6__uncultured organism | 5 |
| 16 | 11 | 0.20 | 0.05 | D_0__Bacteria;D_1__Firmicutes;D_2__Clostridia;D_3__Clostridiales;D_4__Family XIII;D_5__Mogibacterium;__ | 5 |
| 11 | 6 | 0.10 | 0.08 | D_0__Bacteria;D_1__Bacteroidetes;D_2__Bacteroidia;D_3__Bacteroidales;D_4__Prevotellaceae;D_5__Prevotella 7;D_6__Prevotella denticola | 5 |
| 6 | 2 | 0.05 | 0.01 | D_0__Bacteria;D_1__Actinobacteria;D_2__Actinobacteria;D_3__Bifidobacteriales;D_4__Bifidobacteriaceae;D_5__Alloscardovia;D_6__Bifidobacterium longum subsp. longum | 4 |
| 5 | 1 | 0.01 | 0.00 | D_0__Bacteria;D_1__Bacteroidetes;D_2__Bacteroidia;D_3__Bacteroidales;D_4__Prevotellaceae;D_5__Prevotella 2;D_6__unidentified | 4 |
| 5 | 1 | 0.04 | 0.01 | D_0__Bacteria;D_1__Proteobacteria;D_2__Gammaproteobacteria;D_3__Betaproteobacteriales;D_4__Neisseriaceae;D_5__Neisseria;D_6__unidentified | 4 |
| 21 | 17 | 0.53 | 0.70 | D_0__Bacteria;D_1__Fusobacteria;D_2__Fusobacteriia;D_3__Fusobacteriales;D_4__Leptotrichiaceae;D_5__Leptotrichia;D_6__uncultured bacterium | 4 |
| 5 | 1 | 0.02 | 0.00 | D_0__Bacteria;D_1__Bacteroidetes;D_2__Bacteroidia;D_3__Flavobacteriales;D_4__Flavobacteriaceae;D_5__Capnocytophaga;D_6__Capnocytophaga granulosa | 4 |
| 8 | 4 | 0.13 | 0.14 | D_0__Bacteria;D_1__Actinobacteria;D_2__Actinobacteria;D_3__Bifidobacteriales;D_4__Bifidobacteriaceae;D_5__Scardovia;D_6__unidentified | 4 |
| 5 | 1 | 0.01 | 0.00 | D_0__Bacteria;D_1__Bacteroidetes;D_2__Bacteroidia;D_3__Bacteroidales;D_4__Prevotellaceae;D_5__Prevotella 6;__ | 4 |
| 10 | 6 | 0.57 | 0.26 | D_0__Bacteria;D_1__Proteobacteria;D_2__Gammaproteobacteria;D_3__Pasteurellales;D_4__Pasteurellaceae;D_5__Aggregatibacter;D_6__uncultured bacterium | 4 |
| 5 | 1 | 0.03 | 0.02 | D_0__Bacteria;D_1__Epsilonbacteraeota;D_2__Campylobacteria;D_3__Campylobacterales;D_4__Campylobacteraceae;D_5__Campylobacter;D_6__Campylobacter concisus | 4 |
| 17 | 13 | 0.90 | 0.75 | D_0__Bacteria;D_1__Firmicutes;D_2__Negativicutes;D_3__Selenomonadales;D_4__Veillonellaceae;D_5__Megasphaera;D_6__unidentified | 4 |
| 11 | 7 | 0.05 | 0.04 | D_0__Bacteria;D_1__Firmicutes;D_2__Clostridia;D_3__Clostridiales;D_4__Family XIII;D_5__[Eubacterium] brachy group;D_6__Eubacterium brachy ATCC 33089 | 4 |
| 18 | 14 | 0.60 | 0.59 | D_0__Bacteria;D_1__Firmicutes;D_2__Clostridia;D_3__Clostridiales;D_4__Family XI;D_5__Parvimonas;__ | 4 |
| 12 | 8 | 0.11 | 0.23 | D_0__Bacteria;D_1__Fusobacteria;D_2__Fusobacteriia;D_3__Fusobacteriales;D_4__Leptotrichiaceae;D_5__Leptotrichia;D_6__Leptotrichia wadei F0279 | 4 |
| 19 | 15 | 0.38 | 0.39 | D_0__Bacteria;D_1__Firmicutes;D_2__Erysipelotrichia;D_3__Erysipelotrichales;D_4__Erysipelotrichaceae;D_5__Solobacterium;__ | 4 |
| 19 | 15 | 0.17 | 0.20 | D_0__Bacteria;D_1__Firmicutes;D_2__Clostridia;D_3__Clostridiales;D_4__Lachnospiraceae;D_5__Lachnoanaerobaculum;D_6__uncultured bacterium | 4 |
| 14 | 11 | 1.37 | 0.89 | D_0__Bacteria;D_1__Bacteroidetes;D_2__Bacteroidia;D_3__Bacteroidales;D_4__Prevotellaceae;D_5__Prevotella;D_6__Prevotella pallens | 3 |
| 6 | 3 | 0.18 | 0.03 | D_0__Bacteria;D_1__Bacteroidetes;D_2__Bacteroidia;D_3__Bacteroidales;D_4__Prevotellaceae;D_5__Prevotella;D_6__Prevotella aurantiaca JCM 15754 | 3 |
| 18 | 15 | 9.50 | 4.54 | D_0__Bacteria;D_1__Bacteroidetes;D_2__Bacteroidia;D_3__Bacteroidales;D_4__Prevotellaceae;D_5__Prevotella 7;D_6__Prevotella melaninogenica | 3 |
| 10 | 7 | 0.05 | 0.04 | D_0__Bacteria;D_1__Actinobacteria;D_2__Coriobacteriia;D_3__Coriobacteriales;D_4__Atopobiaceae;D_5__Atopobium;__ | 3 |
| 9 | 6 | 0.06 | 0.41 | D_0__Bacteria;D_1__Actinobacteria;D_2__Actinobacteria;D_3__Micrococcales;D_4__Micrococcaceae;D_5__Rothia;D_6__uncultured organism | 3 |
| 19 | 16 | 3.57 | 2.22 | D_0__Bacteria;D_1__Proteobacteria;D_2__Gammaproteobacteria;D_3__Betaproteobacteriales;D_4__Neisseriaceae;D_5__Neisseria;D_6__uncultured bacterium | 3 |
| 21 | 18 | 2.79 | 4.05 | D_0__Bacteria;D_1__Firmicutes;D_2__Bacilli;D_3__Bacillales;D_4__Family XI;D_5__Gemella;__ | 3 |
| 8 | 5 | 1.20 | 0.12 | D_0__Bacteria;D_1__Proteobacteria;D_2__Gammaproteobacteria;D_3__Pasteurellales;D_4__Pasteurellaceae;D_5__Actinobacillus;__ | 3 |
| 8 | 5 | 0.02 | 0.07 | D_0__Bacteria;D_1__Firmicutes;D_2__Negativicutes;D_3__Selenomonadales;D_4__Veillonellaceae;__;__ | 3 |
| 20 | 17 | 4.01 | 3.57 | D_0__Bacteria;D_1__Bacteroidetes;D_2__Bacteroidia;D_3__Bacteroidales;D_4__Prevotellaceae;D_5__Prevotella 7;__ | 3 |
| 6 | 3 | 0.17 | 0.05 | D_0__Bacteria;D_1__Proteobacteria;D_2__Gammaproteobacteria;D_3__Pasteurellales;D_4__Pasteurellaceae;D_5__Aggregatibacter;__ | 3 |
| 6 | 4 | 0.04 | 0.01 | D_0__Bacteria;D_1__Actinobacteria;D_2__Coriobacteriia;D_3__Coriobacteriales;D_4__Atopobiaceae;D_5__Atopobium;D_6__uncultured Actinomyces sp. | 2 |
| 6 | 4 | 0.06 | 0.02 | D_0__Bacteria;D_1__Firmicutes;D_2__Clostridia;D_3__Clostridiales;D_4__Peptococcaceae;D_5__Peptococcus;__ | 2 |
| 18 | 16 | 0.31 | 0.73 | D_0__Bacteria;D_1__Firmicutes;D_2__Clostridia;D_3__Clostridiales;D_4__Lachnospiraceae;D_5__Oribacterium;D_6__Oribacterium sinus | 2 |
| 16 | 14 | 0.09 | 0.28 | D_0__Bacteria;D_1__Firmicutes;D_2__Clostridia;D_3__Clostridiales;D_4__Lachnospiraceae;D_5__Catonella;D_6__uncultured bacterium | 2 |
| 20 | 18 | 6.27 | 3.21 | D_0__Bacteria;D_1__Firmicutes;D_2__Bacilli;D_3__Lactobacillales;D_4__Streptococcaceae;D_5__Streptococcus;D_6__Streptococcus salivarius subsp. thermophilus | 2 |
| 4 | 2 | 0.01 | 0.00 | D_0__Bacteria;D_1__Fusobacteria;D_2__Fusobacteriia;D_3__Fusobacteriales;D_4__Leptotrichiaceae;D_5__Leptotrichia;D_6__Leptotrichia sp. oral clone EI022 | 2 |
| 7 | 5 | 0.10 | 0.02 | D_0__Bacteria;D_1__Proteobacteria;D_2__Gammaproteobacteria;D_3__Pasteurellales;D_4__Pasteurellaceae;__;__ | 2 |
| 7 | 9 | 0.03 | 0.15 | D_0__Bacteria;D_1__Firmicutes;D_2__Clostridia;D_3__Clostridiales;D_4__Ruminococcaceae;D_5__Ruminococcaceae UCG-014;__ | 2 |
| 9 | 7 | 0.08 | 0.11 | D_0__Bacteria;D_1__Firmicutes;D_2__Clostridia;D_3__Clostridiales;D_4__Lachnospiraceae;D_5__Oribacterium;D_6__Oribacterium parvum ACB1 | 2 |
| 10 | 8 | 0.49 | 0.33 | D_0__Bacteria;D_1__Fusobacteria;D_2__Fusobacteriia;D_3__Fusobacteriales;D_4__Leptotrichiaceae;D_5__Leptotrichia;D_6__Leptotrichia sp. oral clone FP036 | 2 |
| 12 | 10 | 0.06 | 0.06 | D_0__Bacteria;D_1__Firmicutes;D_2__Negativicutes;D_3__Selenomonadales;D_4__Veillonellaceae;D_5__Dialister;__ | 2 |
| 13 | 12 | 0.36 | 0.36 | D_0__Bacteria;D_1__Actinobacteria;D_2__Actinobacteria;D_3__Micrococcales;D_4__Micrococcaceae;D_5__Rothia;__ | 1 |
| 4 | 5 | 0.01 | 0.01 | D_0__Bacteria;D_1__Firmicutes;D_2__Clostridia;D_3__Clostridiales;D_4__Lachnospiraceae;D_5__Shuttleworthia;__ | 1 |
| 6 | 5 | 0.19 | 0.10 | D_0__Bacteria;D_1__Firmicutes;D_2__Bacilli;D_3__Lactobacillales;D_4__Lactobacillaceae;D_5__Lactobacillus;__ | 1 |
| 21 | 20 | 2.62 | 2.38 | D_0__Bacteria;D_1__Firmicutes;D_2__Bacilli;D_3__Lactobacillales;D_4__Carnobacteriaceae;D_5__Granulicatella;__ | 1 |
| 7 | 6 | 0.02 | 0.02 | D_0__Bacteria;D_1__Firmicutes;D_2__Negativicutes;D_3__Selenomonadales;D_4__Veillonellaceae;D_5__Selenomonas;D_6__uncultured bacterium | 1 |
| 5 | 6 | 0.07 | 0.09 | D_0__Bacteria;D_1__Firmicutes;D_2__Clostridia;D_3__Clostridiales;D_4__Lachnospiraceae;D_5__Johnsonella;D_6__uncultured bacterium | 1 |
| 19 | 18 | 4.94 | 2.80 | D_0__Bacteria;D_1__Proteobacteria;D_2__Gammaproteobacteria;D_3__Pasteurellales;D_4__Pasteurellaceae;D_5__Haemophilus;__ | 1 |
| 4 | 5 | 0.02 | 0.04 | D_0__Bacteria;D_1__Firmicutes;D_2__Clostridia;D_3__Clostridiales;D_4__Ruminococcaceae;D_5__Ruminococcaceae UCG-014;D_6__Clostridiales bacterium oral taxon 075 | 1 |
| 4 | 5 | 0.04 | 0.03 | D_0__Bacteria;D_1__Firmicutes;D_2__Clostridia;D_3__Clostridiales;D_4__Lachnospiraceae;D_5__Butyrivibrio 2;D_6__Eubacterium sp. oral clone GI038 | 1 |
| 3 | 3 | 0.04 | 0.16 | D_0__Bacteria;D_1__Tenericutes;D_2__Mollicutes;D_3__Mollicutes RF39;D_4__uncultured bacterium;D_5__uncultured bacterium;D_6__uncultured bacterium | 0 |
| 3 | 3 | 0.02 | 0.02 | D_0__Bacteria;D_1__Fusobacteria;D_2__Fusobacteriia;D_3__Fusobacteriales;D_4__Leptotrichiaceae;D_5__Leptotrichia;D_6__Leptotrichia buccalis C-1013-b | 0 |
| 3 | 3 | 0.11 | 0.01 | D_0__Bacteria;D_1__Firmicutes;D_2__Clostridia;D_3__Clostridiales;D_4__Lachnospiraceae;__;__ | 0 |
| 5 | 5 | 0.03 | 0.03 | D_0__Bacteria;D_1__Firmicutes;D_2__Clostridia;D_3__Clostridiales;D_4__Lachnospiraceae;D_5__Lachnoanaerobaculum;__ | 0 |
| 21 | 21 | 9.62 | 8.29 | D_0__Bacteria;D_1__Firmicutes;D_2__Negativicutes;D_3__Selenomonadales;D_4__Veillonellaceae;D_5__Veillonella;__ | 0 |
| 10 | 10 | 0.05 | 0.19 | D_0__Bacteria;D_1__Bacteroidetes;D_2__Bacteroidia;D_3__Bacteroidales;D_4__Prevotellaceae;D_5__Prevotella;D_6__Prevotella nigrescens | 0 |
| 21 | 21 | 3.92 | 5.89 | D_0__Bacteria;D_1__Fusobacteria;D_2__Fusobacteriia;D_3__Fusobacteriales;D_4__Fusobacteriaceae;D_5__Fusobacterium;__ | 0 |
| 5 | 5 | 0.05 | 0.03 | D_0__Bacteria;D_1__Bacteroidetes;D_2__Bacteroidia;D_3__Bacteroidales;D_4__Prevotellaceae;D_5__Prevotella 7;D_6__Prevotella veroralis DSM 19559 = JCM 6290 | 0 |
| 21 | 21 | 23.94 | 39.08 | D_0__Bacteria;D_1__Firmicutes;D_2__Bacilli;D_3__Lactobacillales;D_4__Streptococcaceae;D_5__Streptococcus;__ | 0 |

**Table S4:** Two groups of taxa (light blue and dark blue) that have stronger co-correlations with another taxon than with total load. Significance values for all correlations and co-correlations were *P* < 0.001.

| **Taxon 1** | **Taxon 2** | **Co-Correlation** | **Correlation with  Total Load** | **Difference** | **Biological Link** |
| --- | --- | --- | --- | --- | --- |
| *Alloprevotella* | *Prevotella* | 0.73 | 0.30 | 0.43 | Tertiary plaque biofilm colonizers. Metabolize same byproduct of primary colonizers^5, 6^. |
| *Prevotella 6* | *Prevotella 7* | 0.83 | 0.43 | 0.39 |  |
| *Porphyromonas* | *Prevotella* | 0.74 | 0.36 | 0.38 |  |
| *Prevotella* | *Prevotella 7* | 0.82 | 0.50 | 0.32 |  |
| *Megasphaera* | *Solobacterium* | 0.69 | 0.43 | 0.26 | Not Known |
| *Solobacterium* | *Oribacterium* | 0.80 | 0.55 | 0.25 |  |
| *Leptotrichia* | *Oribacterium* | 0.81 | 0.60 | 0.20 |  |
| *Atopobium* | *Solobacterium* | 0.78 | 0.61 | 0.17 |  |

**Detailed Contributions of Non-corresponding Authors**

Jacob T. Barlow

- Major contributor to idea of applying absolute taxon load via quant seq to small intestinal aspirate samples
- Minor contributor to selection of samples during study design
- Major contributor to library prep for quant-seq
- Performed all digital PCR experiments
- Developed and performed analysis for all figures
- Generated all figures
- Wrote original draft of paper
- Managed all data and code archiving in public repositories

Anna E. Romano

- Major contributor to library prep for quant-seq
- Reviewed and edited original draft of paper

Gabriela Leite

- Major contributor to idea of applying absolute taxon load via quant seq to small intestinal aspirate samples
- Major contributor to selection of samples during study design
- Major contributor to patient data curation
- Developed method for DNA extraction from duodenal aspirates
- Minor contributor to DNA extraction
- Reviewed and edited original draft of paper

Shreya Celly

- Performed all duodenal bacterial cultures and samples processing prior DNA extraction
- Major contributor to DNA extraction

Rashin Sedighi

- Major contributor to patient recruitment
- Major contributor to blood and saliva collection

Christine Chang

- Major contributor to patient recruitment
- Major contributor to blood and saliva collection

Ali Rezaie

- Supervision of patient data curation
- Major contributor to patient upper endoscopy procedure and sample collection from duodenum
- Reviewed and edited original draft of paper

Ruchi Mathur

- Supervision of patient recruitment
- Reviewed and edited original draft of paper

Mark Pimentel

- Supervision of the study
- Major contributor to patient upper endoscopy procedure and sample collection from duodenum
- Reviewed and edited original draft of paper

**Supplementary References**

1. Elazhary, M.A., Saheb, S.A., Roy, R.S. & Lagacé, A. A simple procedure for the preliminary identification of aerobic gram negative intestinal bacteria with special reference to the Enterobacteriaceae. *Canadian journal of comparative medicine : Revue canadienne de medecine comparee* **37**, 43-46 (1973).

2. Ruoff, K.L. Miscellaneous Catalase-Negative, Gram-Positive Cocci: Emerging Opportunists. *J. Clin. Microbiol.* **40**, 1129 (2002).

3. Bogatyrev, S.R., Rolando, J.C. & Ismagilov, R.F. Self-reinoculation with fecal flora changes microbiota density and composition leading to an altered bile-acid profile in the mouse small intestine. *Microbiome* **8**, 19 (2020).

4. Leite, G.G.S. et al. Mapping the Segmental Microbiomes in the Human Small Bowel in Comparison with Stool: A REIMAGINE Study. *Dig. Dis. Sci.* **65**, 2595-2604 (2020).

5. Marcotte, H. & Lavoie, M.C. Oral microbial ecology and the role of salivary immunoglobulin A. *Microbiology and molecular biology reviews : MMBR* **62**, 71-109 (1998).

6. Hojo, K., Nagaoka, S., Ohshima, T. & Maeda, N. Bacterial Interactions in Dental Biofilm Development. *J. Dent. Res.* **88**, 982-990 (2009).
